# Telomere bacteriophages are widespread and equip their bacterial hosts with potent interbacterial weapons

**DOI:** 10.1126/sciadv.adt1627

**Published:** 2025-04-30

**Authors:** Sally M. H. Byers, Andrea Rocker, To N. T. Nguyen, Natalia C. Rosas, George Taiaroa, Kher Shing Tan, Yan Li, Jonathan J. Wilksch, Joel R. Steele, Ralf B. Schittenhelm, Rhys A. Dunstan, Francesca L. Short, Trevor Lithgow

**Affiliations:** ^1^ Infection Program, Biomedicine Discovery Institute and Department of Microbiology, Monash University, Clayton 3800, Australia.; ^2^ Centre to Impact AMR, Monash University, Clayton 3800, Australia.; ^3^ Department of Microbiology and Immunology, The Peter Doherty Institute, The University of Melbourne, Parkville 3052, Australia.; ^4^ Monash Proteomics & Metabolomics Platform, Monash University, Clayton 3800, Australia.

## Abstract

Bacteriophages (phages) are viruses that can kill bacteria, thereby editing and shaping microbial communities. The telomere phages are a curious form using telomere-like structures to replicate their genomes as linear extrachromosomal elements. Here, we find that telomere phages are widely distributed in bacteria, being highly prevalent in *Klebsiella* species. We establish a model system to investigate telomere phage biology by isolating the virions of telomere phages and infecting naïve strains to create isogenic lines with and without a phage. We find that only a small set of telomere phage proteins is expressed in phage-host cells, including a toxin—the telocin—that kills other *Klebsiella* strains. We identify and validate a set of telocins in the genomes of other prevalent *Klebsiella* telomere phages. Thus, telomere phages are widespread elements encoding diverse antibacterial weapons and we discuss the prospect of using telocins for precision editing of microbial populations.

## Teaser

*Klebsiella* host strains infected with telomere phages can grow to be the dominant lineage in mixed populations.

## INTRODUCTION

The *Klebsiella pneumoniae* species complex (*Klebsiella* spp.) is a collection of closely related species of bacteria, which show a relatively recent evolutionary divergence (*1*–*3*). The *Klebsiella* spp. are found as dominant members in agricultural and environmental niches on plants and in water and, as a result, in invertebrate and vertebrate guts (*4*–*12*). Notoriously, the signature species *K. pneumoniae* is one of six ESKAPE pathogens for which new therapeutic strategies are of urgent priority (*13*, *14*). The past decade has seen a rise in drug-resistant *Klebsiella* (*14*), the emergence of community-acquired, hypervirulent *Klebsiella* strains that cause disease in healthy individuals (*15*, *16*), and the evolution of convergent strains displaying both hypervirulence and antimicrobial resistance (*17*). The evolution of these strains is attributable in part to the extensive array of mobile genetic elements that circulate within *Klebsiella* populations, including but not limited to circular (*18*) and linear (*19*) plasmids, integrative chromosomal elements (*20*, *21*), and temperate bacteriophages (*22*, *23*). In a recent study, we analyzed the evolution of a strain of carbapenem-resistant *Klebsiella* spp., where whole-genome sequencing revealed it to have a megaplasmid that contributed to the evolution of antimicrobial resistance, and a previously undescribed telomere phage we introduce herein as NAR688 (*24*).

Telomere phages have long been considered a rare biological curiosity. Somewhat akin to both temperate phages and linear plasmids, telomere phages have a unique means of DNA replication (*25*, *26*) multiplying as a linear, double-stranded DNA element capped on either end with hairpin structures that are analogous—but not homologous—to the telomeres that cap eukaryotic chromosomes (*27*, *28*). Unlike canonical temperate phages, telomere phages do not integrate into the bacterial chromosome during their life cycle, instead being maintained as independent genetic entities in the host cytoplasm during vegetative growth. Thus, these elements can be propagated vertically as if they were plasmids and horizontally as bacteriophages. Previously characterized telomere phages include N15 of *Escherichia coli* (*28*, *29*), PhiKO2 of *Klebsiella oxytoca* (*30*), and PY54 of *Yersinia enterocolitica* (*31*). The genomes of N15, PhiKO2, and PY54 are similar in size, replicative mechanism, and genetic arrangement and exhibit homology in the genes involved in the maintenance of the linear plasmid form. However, little is known about the prevalence of these phages in their respective host bacteria or about the roles they play in the biology of their bacterial hosts.

Serendipitously, we found and thus sought to characterize the *Klebsiella* telomere phage NAR688, with a view to understanding its role in *Klebsiella* biology. Through genomic analyses, further NAR688-like telomere phages were identified in more than 10% of surveyed *Klebsiella* genomes, representing a substantial proportion of the *Klebsiella* population. We successfully isolated NAR688 virions for characterization by electron microscopy and used them to create isogenic strains with and without the telomere phage. A proteomic analysis of these strains revealed that NAR688 encodes a bacteriocin type toxin, telocin A (TelA), which enables killing of cocultured TelA-sensitive strains. Four related telomere phages were identified as encoding four distinct telocins (B, C, D, and E) within the equivalent locus of their genomes. The results suggest that telomere phages can promote competition between *Klebsiella* strains by facilitating the transfer and evolution of *Klebsiella*-targeting toxins.

## RESULTS

### Telomere phages are prevalent in *Klebsiella* hosts

**Fig. 1.**
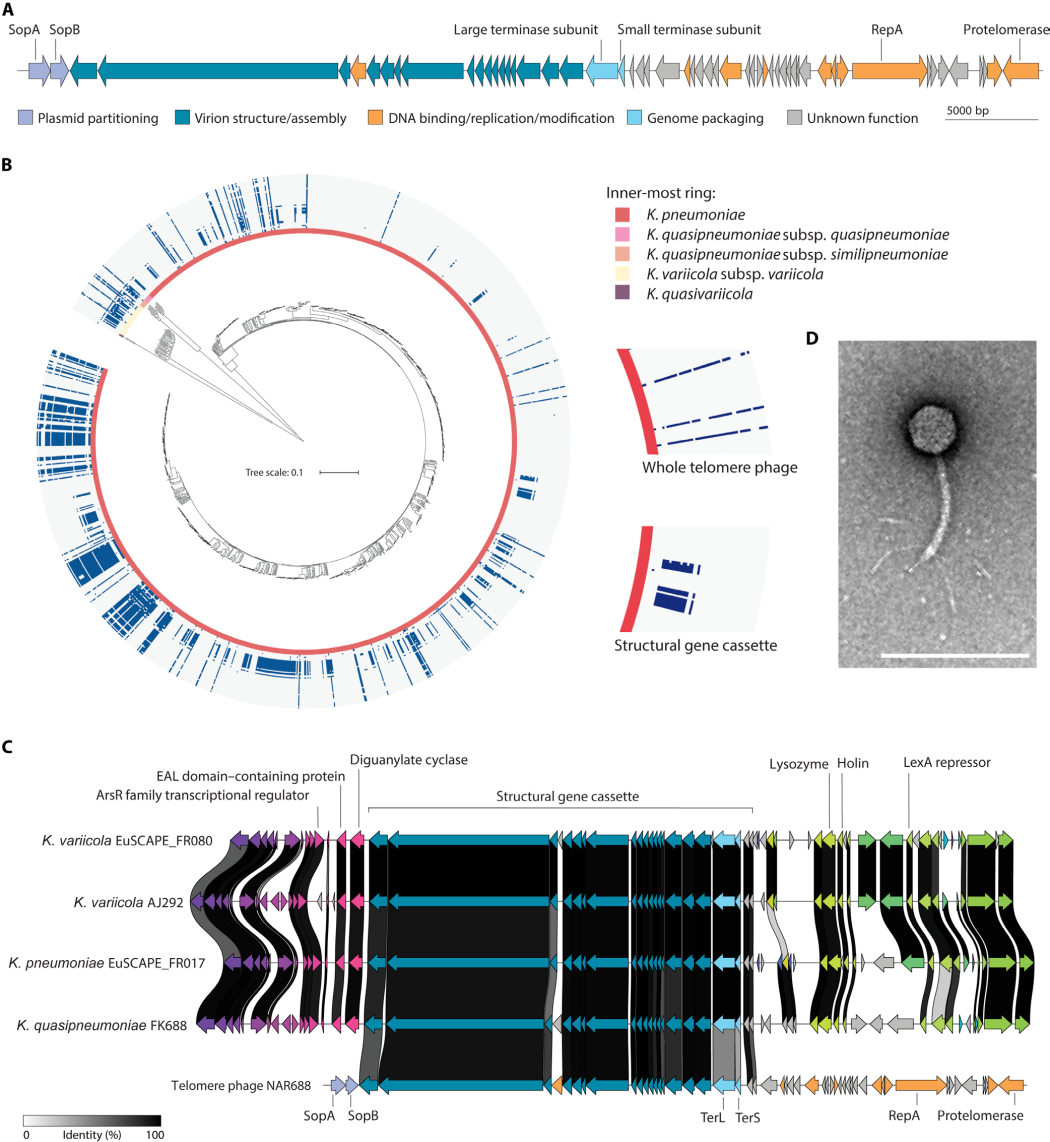
Telomere phage NAR688. (**A**) Genome sequence of NAR688 (CP072507.2) annotated with color coding of the predicted phage protein function. The gene encoding the diagnostic protelomerase is shaded orange. The detail in the annotated genome is also provided (table S1). (**B**) Distribution of NAR688 gene hits (90% identity over 70% sequence coverage) across the *K. pneumoniae* species complex dataset, a curated and representative selection of 1309 complete genomes of five species: *K. pneumoniae*, *K. quasipneumoniae* subsp*. quasipneumoniae*, *K. quasipneumoniae* subsp. *similipneumoniae*, *K. variicola* subsp. *variicola*, and *Klebsiella quasivariicola*. These are indicated around the inner circle. The absence (gray) or presence (blue) of TelA phage–like genes is shown. Inset: Likely SGCs are indicated by the partial shading of blue for the structural genes only, while a near-complete set of genes spanning the ring indicates the presence of what may be an intact telomere phage. (**C**) Alignment of NAR688 genome and chromosomal regions encoding SGCs in two exemplar sequences from the pangenome dataset: *K. variicola* EuSCAPE_FR080 (17870_42SCcontig000002) and *K. pneumoniae* EuSCAPE_FR017 (UKHK01000001.1), the NAR688-susceptible strain *K. variicola* AJ292, and the original source of phage NAR688, *K. quasipneumoniae* FK688. Alignment produced using clinker (*63*). (**D**) After treating *Klebsiella* FK688 with mitomycin C, cell lysates were applied to CsCl gradients to purify NAR688 virions for imaging by transmission electron microscopy. Scale bar, 200 nm. Further image analysis is documented in fig. S1.

Analysis of *Klebsiella quasipneumoniae* FK688 (*24*) long-read sequence data revealed that the strain has a linear, extrachromosomal genetic element we call NAR688. Annotation of this genetic element (table S1) revealed 55 open-reading frames, encompassing genes required for phage genome replication and virion production, such as the genome-packaging motor subunits TerL and TerS, as well as hallmark telomere phage genes: plasmid-partitioning proteins SopA and SopB and the enzyme responsible for maintenance of DNA in the linear-plasmid form, protelomerase (Fig. 1A).

To assess the prevalence of NAR688-like telomere phages in *Klebsiella*, we constructed a pangenome dataset representing the *K. pneumoniae* species complex using a selection of publicly available *Klebsiella* genomes compiled as a well-defined representative collection (data S1) (*32*). NAR688 coding sequences were used to query the pangenome for related sequences with a stringent threshold of ≥90% nucleotide identity over 70% gene coverage for a hit (data S1), and each NAR688 gene was mapped onto a phylogenetic tree of genomes in the dataset. The resulting phylogram (Fig. 1B) shows that NAR688-like phages are broadly distributed in the species complex. An elongated line of identity is apparent in strains that would have a NAR688-related telomere phage. In addition, in many strains, a truncated segment of identity indicated what was found to be a gene cassette where the genes were found to correspond to those encoding the 17 structural proteins of NAR688 virions; we thus refer to them as structural gene cassettes (SGCs) (Fig. 1C). Thus, in terms of prevalence, (i) telomere phages were found to be present in 11.6% of the surveyed *Klebsiella* genomes, and (ii) there is a suggested historical presence of telomere phages from the chromosomal integration of telomere phage structural genes.

Interrogation of the SGCs revealed that these cassettes are situated within the bacterial chromosome and are flanked by the same genes across different strains (Fig. 1C). The genes encoding the terminase subunits were found to differ between telomere phages and SGCs, but to be highly conserved within each group, consistent with a hypothesis that the SGCs derive from a single integration event or a few integration events of an ancestral NAR688-like phage preceding *Klebsiella* divergence. The conservation of these SGCs in at least 14.7% of *Klebsiella* chromosomes (data S1) is notable.

### A genetic system to study telomere phage impacts on host cell biology

To obtain an enriched NAR688 phage preparation for imaging and infection studies, we induced the assembly of NAR688 virions by mitomycin C treatment of host strain FK688 and then purified the released phage virions by centrifugation on cesium chloride gradients (fig. S1A). The purified preparation was visualized by negative stain transmission electron microscopy. The NAR688 virions have 133-nm-long, flexible, noncontractile tails with a central tail spike and have multiple long tail fibers emanating from the tail (Fig. 1D and fig. S1B). To assess the bacterial host range, samples of purified phage were screened against a collection of 53 *Klebsiella* strains (table S2). Evidence of phage infection was observed on four of these strains (fig. S1C). Among these, clearance zones on *Klebsiella* strains AJ027 and AJ292 exhibited abundant regrowth, and cells from within the regrowth zones on each strain were passaged. Whole-genome sequencing confirmed that NAR688 had taken up residence within each strain, forming the host-phage strains AJ027ø688 and AJ292ø688. Colony polymerase chain reaction (PCR) using NAR688-specific primers showed that over 10 successive days of passaging, the phage is stably maintained in these strains (fig. S1D), and growth curves comparing NAR688-infected strains (AJ027ø688 and AJ292ø688) and isogenic naïve strains (AJ027 and AJ292) showed that the phage carriage does not affect the growth rate of infected strains (fig. S1E).

### A toxin-immunity protein pair is expressed in the host cell system

**Fig. 2.**
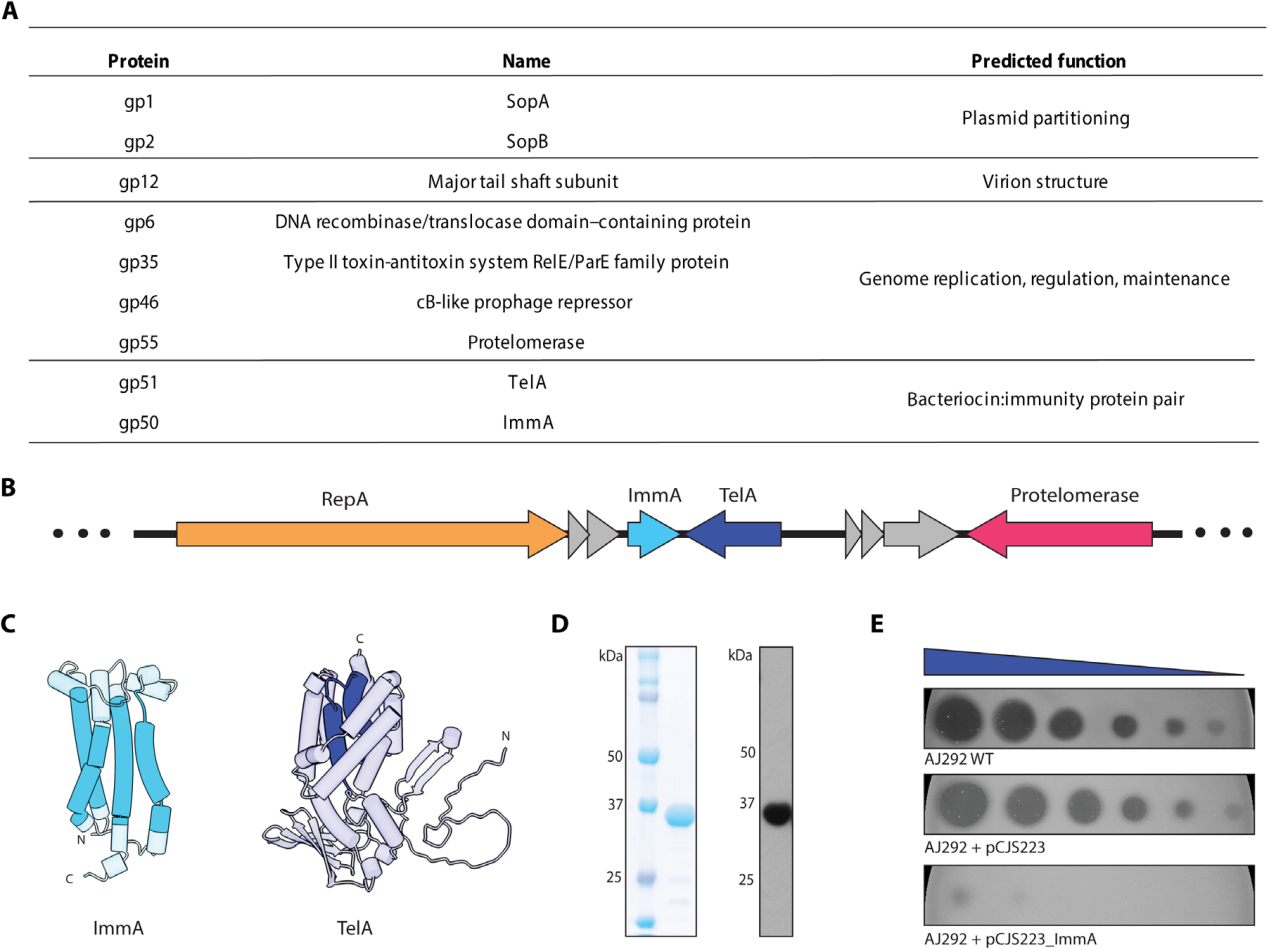
TelA is an antibacterial toxin that can be inhibited by the inner membrane protein ImmA. (**A**) Proteins detected by mass spectrometric assessment of AJ292ø688 cells grown to the midlog phase. Predicted functions, based on genome annotation of phage NAR688, are indicated for each of the phage-encoded proteins detected in the proteome. (**B**) Diagrammatic representation of the TelA-ImmA locus between the genes encoding the origin-binding protein (RepA) and protelomerase. Genes in gray have an unknown function. (**C**) AlphaFold2 predictions of the structures of ImmA and TelA, with predicted transmembrane regions (i.e., hydrophobic α helices) of each protein shown in shades of solid blue. (**D**) Purification of His-tagged TelA as documented on SDS-PAGE using Coomassie blue staining to demonstrate purity and immunoblotting with anti-His_6_ to identify the protein as TelA. (**E**) Serial dilutions of purified TelA were spotted onto lawns of *Klebsiella* AJ292, *Klebsiella* AJ292 + pCJS223, or *Klebsiella* AJ292 + pCJS223_ImmA. The 10-fold dilutions start at ~3.6 μg of TelA. The TelA-immunity protein ImmA is an inner membrane protein (fig. S6). WT, wild type.

We sought to identify phage-encoded proteins expressed during vegetative growth of a host strain. Isogenic strains with or without the telomere phage (i.e., AJ292ø688 and AJ292, respectively) were grown to the midlog phase of culture and harvested for proteomic analysis (see the Materials and Methods). Only nine proteins of NAR688 origin were detected in the AJ292ø688 population (Fig. 2A). Six of these proteins are known or hypothesized to be involved in either the regulation and maintenance of the telomere bacteriophage genome (protelomerase, gp6, gp35, and gp46) or DNA segregation during cell division (SopA and SopB). Only one structural protein (annotated as the “tail shaft subunit”) representing the major tail-tube protein (*33*) was detected. Last, two other proteins of unknown function were detected, and these were observed to be encoded by genes in a small gene cassette (Fig. 2B). Subsequent sequence- and structure-based analyses suggested that the protein we designate as TelA has homology to a group of toxins called pore-forming bacteriocins (Fig. 2C and fig. S2), while the other that we designate as ImmA has homology to proteins that inhibit the activity of cognate toxins (Fig. 2C and fig. S3) (*34*, *35*).

To test whether TelA functions as an antibacterial toxin, we expressed and purified a C-terminal hexahistidine (His)–tagged form of the protein (Fig. 2D and fig. S4) and spotted the purified protein onto soft agar overlays of the same 53 *Klebsiella* strains used for phage host-range determination. After overnight incubation, clearance zones of varying intensities were observed on 31 strains, including *Klebsiella* AJ292 (Fig. 2E and fig. S5), indicating that TelA has broad bactericidal activity against *Klebsiella* strains. The predicted immunity protein, ImmA, was shown to confer toxin resistance when expressed in AJ292 cells (Fig. 2E). For its antibacterial activity and genetic origins in a telomere phage, we refer to the toxin as TelA and to its cognate immunity protein as ImmA.

ImmA is predicted to localize to the bacterial inner membrane (see the Materials and Methods), and AlphaFold2 suggests that ImmA has four α helices that are hydrophobic, as would be expected in transmembrane helices (fig. S6A). To test the predicted subcellular location of ImmA, AJ292 cells expressing a His-tagged form of ImmA were subject to sucrose gradient subcellular fractionation and immunoblot analysis. ImmA was found to be enriched in the inner membrane fraction (fig. S6B). The localization of ImmA in the inner membrane would suggest that TelA functions at the inner membrane, consistent with pore-forming toxins (*34*).

### OmpK36, TonB, and ExbB participate in TelA import

**Fig. 3.**
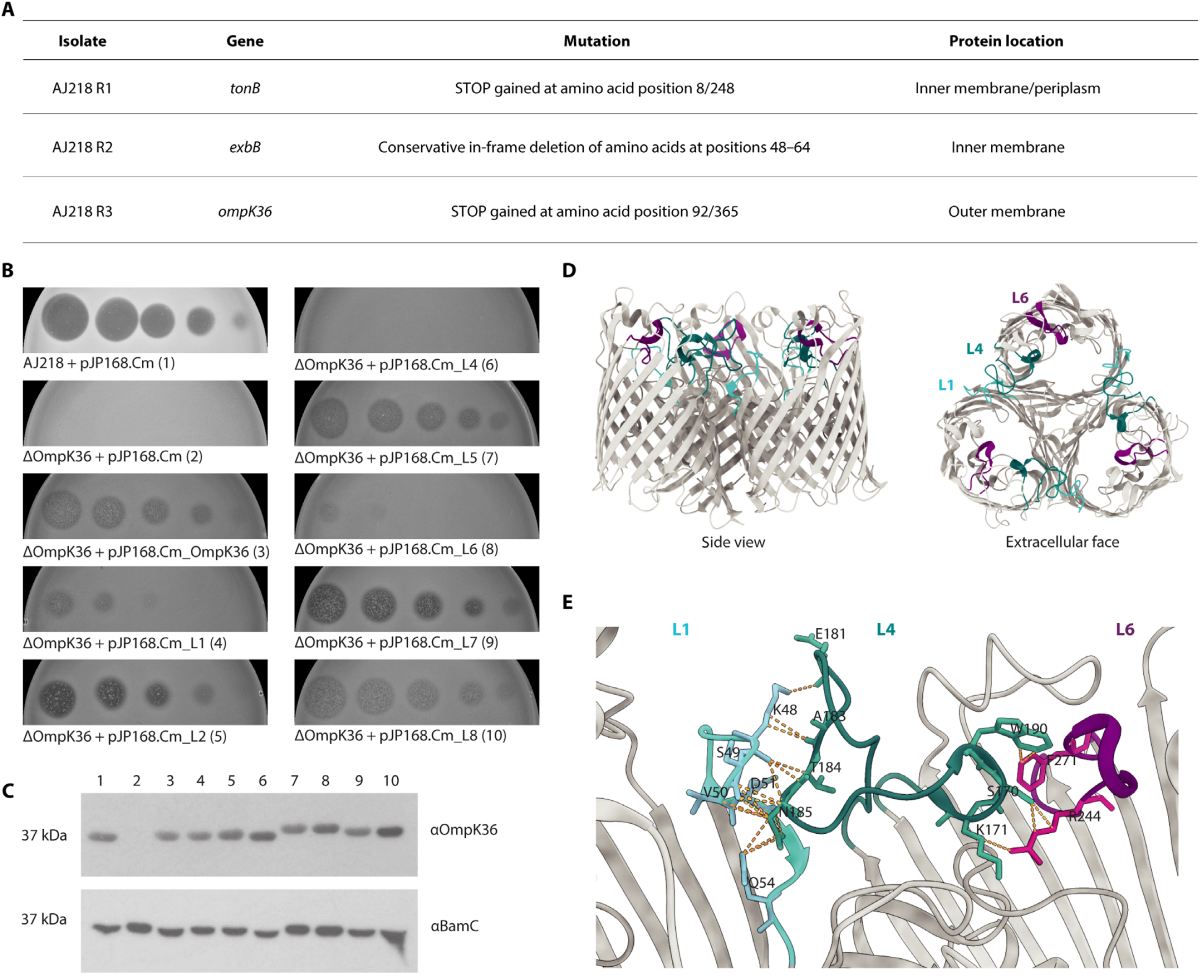
Import of TelA depends on the surfaced-exposed features of receptor OmpK36. (**A**) Mutations in three TelA-resistant AJ218 mutants suggested that *tonB*, *exdB*, and *ompK36* are involved in TelA entry into *Klebsiella*. Further details are shown in fig. S7. (**B**) TelA dilution series spot tested against *K. pneumoniae* AJ218 bearing the pJP168.Cm vector, AJ218∆*ompK36* bearing the pJP168.Cm vector, AJ218∆*ompK36* with OmpK36 expressed from the pJP168.Cm vector, and AJ218∆*ompK36* expressing OmpK36 loop chimeras (L1, L2, and L4 to L8). (**C**) Immunoblot probing for OmpK36 in membranes purified from strains in (B), with lane numbers corresponding to numbers in brackets, to confirm OmpK36 expression in each strain. BamC loading control was included. (**D**) In this representation of the OmpK36 crystal structure (PDB: 6RD3) shown from the side view and extracellular face, loop 1 is colored aqua, loop 4 is colored teal, and loop 6 is colored purple. (**E**) Predicted contacts (orange dashed lines) between OmpK36 loops 1, 4, and 6 in adjacent monomers at the periphery of the trimeric molecule, as modeled by ChimeraX version 1.7.1 (*67*).

To investigate determinants of TelA susceptibility in target cells, breakthrough colonies were isolated from within the clearance zone of *Klebsiella* strain AJ218 treated with TelA in spot test assays. Three colonies were subcultured, and their resistance to the TelA toxin was confirmed through subsequent assays of TelA activity (fig. S7). Whole-genome sequencing of the mutant strains, referred to as R1, R2, and R3, revealed mutations in genes encoding *Klebsiella* proteins TonB, ExbB, and OmpK36, respectively (Fig. 3A). Complementation with the wild-type (WT) genes *tonB*, *exbB*, and *ompK36* into respective mutant strains restored their sensitivity to TelA (fig. S7). In other bacterial systems, TonB and ExbB function together as an energy-transducing system for the import of various bacteriocins (*36*).

OmpK36 is an abundant, trimeric outer membrane porin in *Klebsiella* (*37*). It is a β barrel transmembrane protein exhibiting seven inter–β strand loops that are exposed to the extracellular surface of *Klebsiella* cells. We hypothesized that these extracellular loops could serve as receptor sites to enable TelA to bind the surface of *Klebsiella*. To test this hypothesis, TelA was assayed against a panel of OmpK36 surface-loop mutants (*38*). In each mutant, a single extracellular loop was replaced by the equivalent loop from the related *Klebsiella* porin, OmpK37 (*39*). AJ218 expressing OmpK36 with substitutions of loops 2, 5, 7, and 8 retained wild-type levels of sensitivity to TelA, while sequence substitutions in loops 1 and 6 reduced TelA sensitivity, and the loop 4 substitution rendered cells resistant to TelA (Fig. 3B). The expression of the chimeric porins at approximately wild-type levels was confirmed through immunoblot analysis (Fig. 3C). In the crystal structure of OmpK36 [Protein Data Bank (PDB): 6rd3], loop 4 interacts with loop 6 of the same monomer and with loop 1 of the adjacent monomer (Fig. 3D). We suggest that loops 1 and 6 may play a role in stabilizing the essential binding epitope in loop 4 (Fig. 3, D and E) and propose that OmpK36 is the receptor for TelA import into *Klebsiella*.

### Five telocins, distinguished by host range and structural features

**Fig. 4.**
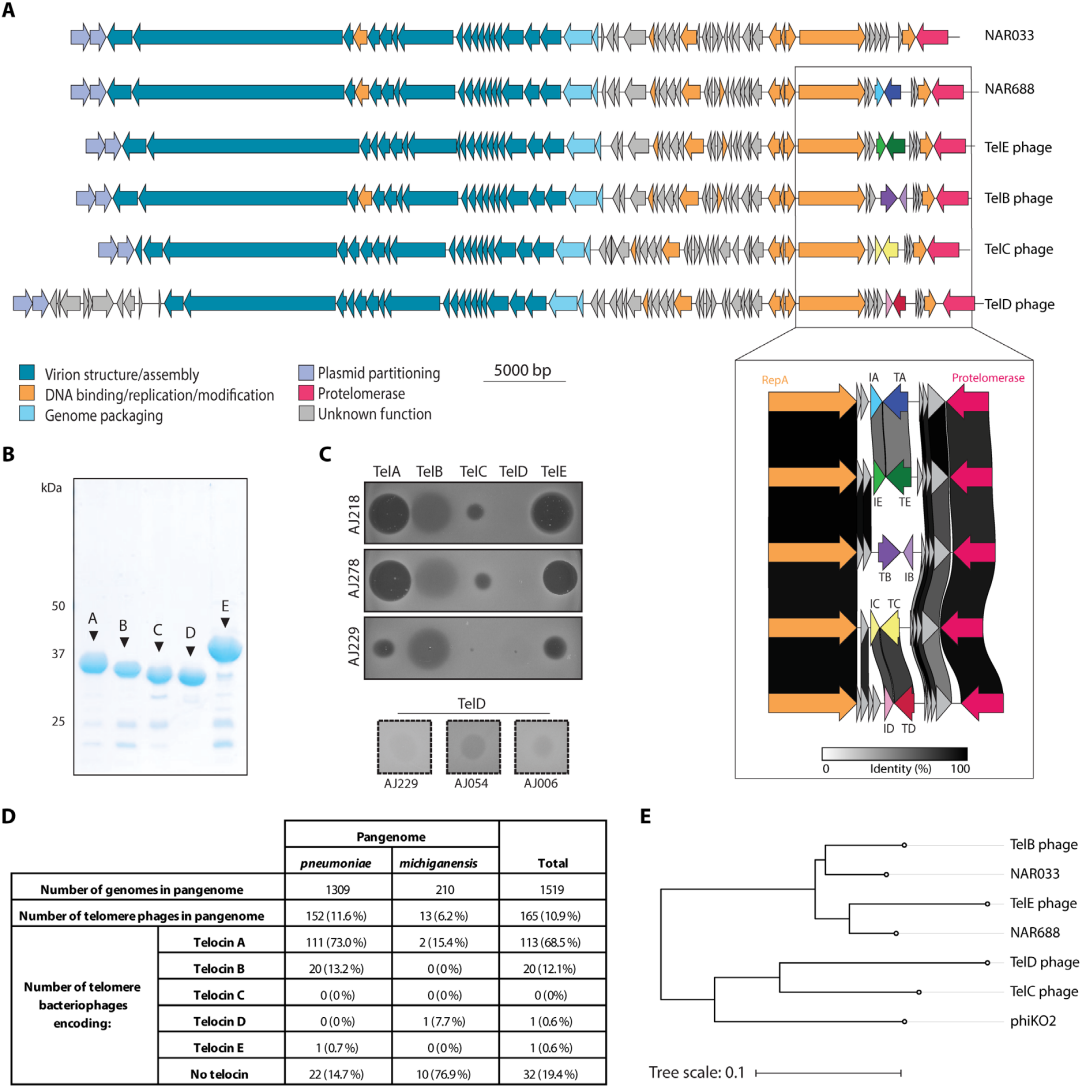
A range of *Klebsiella* telomere phages encodes a set of distinct telocins. (**A**) Schematic of a Tel0 phage (NAR033) and five telocin-encoding telomere phage genomes (NAR688, TelE phage, TelB phage, TelC phage, and TelD phage). Genes are colored according to function. The telocin:immunity protein locus is enlarged, and the amino acid identity of the proteins in this locus, as determined using clinker (*63*), is indicated by shading. TA, TelA; IA, ImmA; TB, TelB; IB, ImmB; TC, TelC; IC, ImmC; TD, TelD; ID, ImmD. (**B**) Purified TelA to TelE were analyzed by SDS-PAGE and staining of the gels with Coomassie blue. (**C**) Spot testing of purified (2 μg) TelA to TelE against three exemplar strains. Inset: spot testing of 20 μg of TelD against three strains. The complete analysis of all strains is shown in fig. S5. (**D**) Prevalence of predicted telomere phages and telocins in the *K. pneumoniae* species complex and *K. michiganensis* pangenomes. *K. pneumoniae* species complex data do not add to 100%; two predicted telomere phages in this dataset encode both TelA and TelB (data S1). (**E**) Phylogenetic tree showing predicted evolutionary relationships (linear representation) between NAR688, NAR033, TelB phage, TelE phage, TelC phage, TelD phage, and *K. oxytoca* telomere phage PhiKO2.

Given the prevalence of NAR688-like telomere phages (Fig. 1B), we sought to determine whether all of these phages carry a TelA-ImmA cassette. Using the phage NAR688 sequence as a query, BLAST searches of the National Center for Biotechnology Information (NCBI) nucleotide collection database of all bacterial genomes identified *Klebsiella* genomes having hallmark telomere phage genes. These were interrogated at the presumptive toxin locus. Four previously undescribed *Klebsiella* telomere phages encoding four distinct telocin gene cassettes were identified; we refer to these as TelB, TelC, TelD, and TelE phages and to their respective telocin:immunity protein pairs as TelB:ImmB, TelC:ImmC, TelD:ImmD, and TelE:ImmE, respectively. In the course of these genome searches, we also identified a telomere phage in *Klebsiella* AJ033 that lacked any telocin; we refer to this phage as NAR033, and we suggest referring more broadly to telomere phages lacking telocins as Tel0 phages. The alignment of these phage genomes suggests that NAR688, TelB phage, TelE phage, and NAR033 are closely related, a key distinction between these genomes being the telocin:immunity locus (Fig. 4A). The TelC phage and TelD phage are closely related to each other but have lower nucleotide identity with the other telomere phages.

To test the antimicrobial activity of these proteins, each of the telocins was expressed as a C-terminal His-tagged form and purified using nickel affinity chromatography and size exclusion chromatography (Fig. 4B and fig. S8, A to D). AlphaFold2 was used to predict structural information (fig. S8, A to D). TelA and TelE share 64% amino acid identity and were found to have structural homology with pore-forming bacteriocins (*40*), while TelB was found to be structurally related to peptidoglycan-degrading enzymes first identified in *Yersinia pestis* to function as bacteriocins (*41*–*43*). TelC and TelD have 86% amino acid identity and structural homology to a recently described peptidoglycan-degrading bacteriocin (*44*). The purified telocins were assayed against 53 *Klebsiella* strains (fig. S5); representative data from three strains are shown as an example (Fig. 4C). TelB exhibited the broadest spectrum, killing 51 of the 53 tested strains, followed by TelA and TelE with activity against 31 and 30 *Klebsiella* strains, respectively. TelC had a comparatively narrow killing spectrum (10 strains), while TelD had activity against only three strains (Fig. 4C and fig. S5). In total, 51 of the 53 tested isolates (96%) were sensitive to some degree to at least one telocin (fig. S5).

To understand the distribution of each telocin in the *K. pneumoniae* species complex genome dataset, phage genome sequences for NAR688, TelC phage, and TelD phage and the amino acid sequences of TelA to TelE were searched against the pangenome dataset to define telocin-encoding telomere phages (fig. S9A and data S1). Most of the NAR688-like putative telomere phages were found to carry a telocin, with TelA being the most prevalent telocin in the dataset, TelB having the second highest prevalence, and TelE being present in only one genome (Fig. 4D and data S1). While TelC and TelD were not present in this historical dataset, TelC was identified in searches of more recently deposited *K. pneumoniae* genomes.

Sequences corresponding to TelD phages were found to be present in a second genome dataset that we compiled for the “*K. michiganensis* pangenome,” a dataset including genomes of other *Klebsiella* species: *Klebsiella michiganensis*, *Klebsiella pasteurii*, and *Klebsiella grimontii* (fig. S9B and data S2). TelA was the only other telocin identified among the putative telomere phages in this dataset (Fig. 4D and data S2). This is consistent with the observation that the NAR688-like phages and TelC/TelD telomere phages cluster separately in a VIPTree-generated phylogeny (*45*). Furthermore, the TelC phage and TelD phage cluster with the previously described *K. oxytoca* telomere phage, PhiKO2 (Fig. 4E), suggesting some shared ancestry in these phages.

**Fig. 5.**
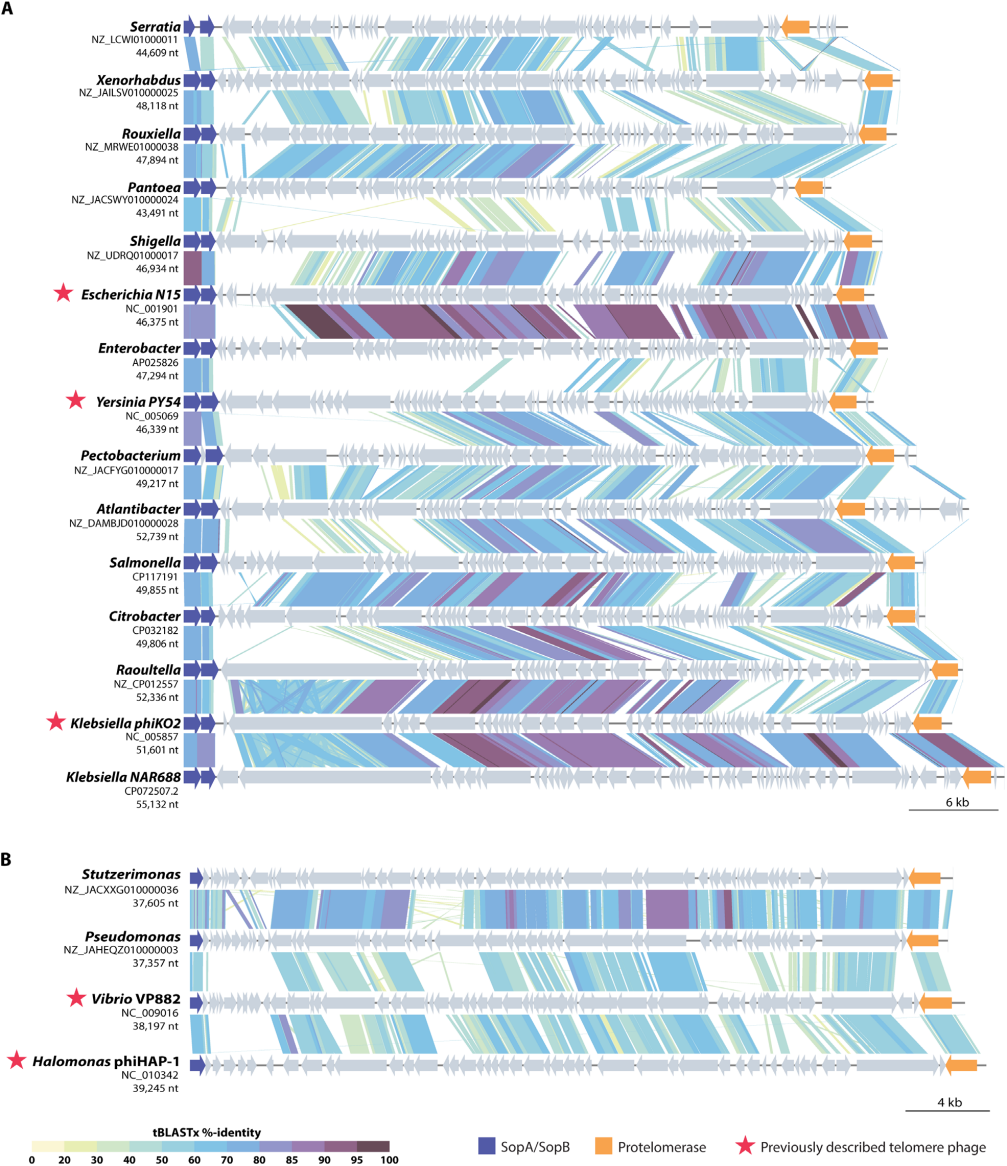
Telomere phages across multiple bacterial genera. Nucleotide sequence alignments are shown for the complete genomes of two phylogenetically distinct clusters of putative telomere phages. Each of these putative telomere phages was identified by sequence encoding a protelomerase enzyme with sequence similarity to that found in phage NAR688. (**A**) Telomere phage sequences that cluster with NAR688, PhiKO2, PY54, and N15. nt, nucleotide. (**B**) Telomere phage sequences that cluster with the putative contractile phages VP882 and phiHAP-1. Alignment was performed with VIPTree (*45*).

To provide insight into whether telomere phages are present in other bacterial groups, we performed iterative searches of the NAR688 protelomerase sequence against non-*Klebsiella* taxa in the NCBI protein database. Analysis of returned proteins and their associated genomes revealed evidence of telomere phages in 13 genera in which they have not been described previously, including many members of Enterobacteriaceae (Fig. 5A). *Pseudomonas* and *Stutzerimonas* telomere phages related to marine telomere phages *Vibrio* VP882 and *Halomonas* PhiHAP-1 were also identified (Fig. 5B). Thus, telomere phages appear far more widespread than had been appreciated.

### Telomere phages confer a competitive advantage against TelA-sensitive strains

**Fig. 6.**
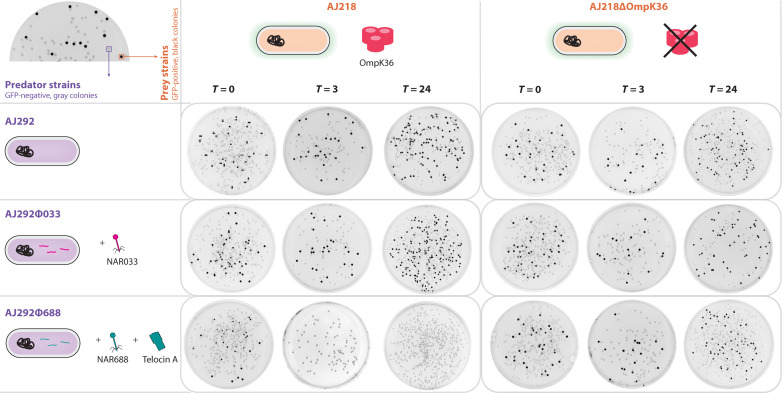
NAR688 confers a competitive advantage to its bacterial host. *Klebsiella* strains AJ292, AJ292ø033, and AJ292ø688 were cocultured with GFP-expressing *Klebsiella* strain AJ218 or AJ218∆*ompK36* over a 24-hour period. Samples of mixed cultures were plated at the time of inoculation (*T* = 0) and at *T* = 3, 6, 9, 12, and 24 hours postinoculation, and the plates were incubated overnight to develop colonies and then imaged under blue light in an Amersham Imager. Representative plates from the *T* = 0-hour, *T* = 3-hour, and *T* = 24-hour time points at dilution factors 10^−3^, 10^−6^, and 10^−6^ (respectively) are shown. Other time points are included in fig. S10. GFP-positive AJ218 and AJ218∆*ompK36* colonies appear black, while GFP-negative AJ292, AJ292ø033, and AJ292ø688 colonies appear gray.

The prevalence of telomere phages is consistent with positive selection for their presence. To assess whether TelA could confer an advantage that could be subject to positive selection, we performed a competitive coculture experiment. *Klebsiella* strains AJ292, AJ292ø033 (which is AJ292 carrying NAR033, a Tel0 phage that does not encode a telocin), and AJ292ø688 (AJ292 carrying phage NAR688) were used as predator strains. As prospective prey strains, we tested the following: (i) AJ218, which is resistant to phage NAR033 and phage NAR688 but sensitive to TelA, and (ii) AJ218∆*ompK36*, an *ompK36* deletion strain (*38*), which is resistant to phage NAR033, phage NAR688, and TelA (Fig. 6). Prey strains were genetically modified to constitutively express green fluorescent protein (GFP), thereby enabling predator and prey strains to be distinguished from each other. Competition events were inoculated in liquid coculture, and samples taken over time were spread on agar plates. The first sample was taken immediately following inoculation (*T* = 0), and further sampling occurred at 3, 6, 9, 12, and 24 hours postinoculation. The resulting colonies were imaged under blue light such that colonies lacking GFP (predator strains) appear gray and colonies expressing GFP (prey strains) appear black.

AJ218 and AJ218∆*ompK36* colonies were detected at all time points in cocultures with AJ292 and AJ292phi033 (Fig. 6 and fig. S10), suggesting that neither the AJ292 strain nor the telocin-negative NAR033 bacteriophage produces factors that eliminate the prey strains. Conversely, in cocultures of AJ292ø688 and AJ218, colony analysis of the samples showed that AJ218 colonies were outcompeted at *T* = 3 onward, suggesting that the TelA-encoding phage provides a competitive advantage to AJ292 cells over AJ218. This effect was not observed using the isogenic TelA-resistant prey strain; AJ218∆*ompK36* was detected at all time points in competition with AJ292ø688 (Fig. 6 and fig. S10). Together, the data are consistent with a model in which TelA enables the telomere phage and host to dominate telocin-sensitive strains.

## DISCUSSION

Bacteriocin is the collective term for toxins that benefit the bacteria that produce them to facilitate colonization or prevent niche invasion by competing bacteria (*35*, *46*, *47*). Bacteriocins are generally named according to the species or genus by which they are produced, such as the colicins of *Escherichia coli* or the klebicins of *Klebsiella.* We propose the use of the term telocin to specify bacteriocins encoded in telomere phage genomes to reflect that, while these proteins have the same ecological function as other bacteriocins, their genetic origins are distinct. For example, TelA has 97% amino acid sequence identity to the previously identified toxins KpneA (*48*) and klebicin E (*49*). Both of these klebicins were identified in *K. pneumoniae* and recombinantly produced to demonstrate their antibacterial activity (*48*, *49*). While any nomenclature system presents issues, we believe that describing as telocins bacteriocins encoded by telomere phages in a gene cassette with a cognate immunity protein is useful for highlighting their mobile nature, which may influence regulatory and other features of these proteins yet to be investigated.

The term “phage-plasmid” has been proposed to cover a diverse class of genetic elements that includes telomere phage N15 and the *Klebsiella* phages presented herein (*50*, *51*). By the definition for phage-plasmids as genetic elements that have both phage-related and plasmid-related genes, telomere phages fit the designator: They encode SopA and SopB proteins for vertical transmission during vegetative growth of the host. While phage-plasmid is a useful categorization for genome-based detection and cataloging, it is also misleading in the sense that telomere phages are phages: We show that their life cycle includes the capacity to package recognizable virions with siphoviridae-type morphology, as well as the capability to infect a naïve host as we demonstrated for phage NAR688. This could be a distinguishing feature of the telomere phages and may not be true for other phage-plasmids, given the extraordinary range of features that Pfeifer *et al.* (*51*) delineated for 780 predicted phage-plasmids in 26 communities across 81 bacterial genera based on weighted gene repertoire relatedness.

A bacterium-telomere phage can also be considered a multispecies individual, and there remains much to understand about the biology and evolutionary advantages of multispecies individuals within mixed populations (*52*, *53*). The methodologies established here provide a means to study these multispecies individuals and the telomere phage-host relationship. The host-telomere phage relationship presented here appears to act under a positive selection pressure, given the high prevalence of telomere phages seen in more than 10% of surveyed *Klebsiella* genomes. Our work also suggests a mutualistic relationship between NAR688 and its *Klebsiella* hosts, mediated through TelA as a weapon of interbacterial competition. The cell benefits from the telocin encoded by NAR688, while NAR688 benefits from the survival of its host cell. Furthermore, the importance of telomere phages in this regard is evidenced by NAR688-like SGCs in the chromosomes of many *Klebsiella* strains lacking resident telomere phages. The function of these gene cassettes is unknown, but their composition is reminiscent of gene cassettes that encode gene transfer agents, phage-like particles that transfer random segments of host DNA between related cells in mixed communities (*54*).

A limitation of this study is that it used reference telomere phage genomes to detect telomere phages among *Klebsiella* genomes, classifying these phages as “NAR688-like” or “TelC/TelD phage–like.” While fit for determining prevalence, this method does not provide a complete representation of telomere phage diversity within these populations. In future work, telomere phage genomes gathered from large datasets could be classified into different “types” based on gene content, with applied metadata enabling us to resolve whether different telomere phage “types” are associated with different species, environments, or other factors. Highly conserved regions among these genomes may allow detection of essential telomere phage genes that contribute to the prevalence of these phages.

Collectively, TelA to TelE display antibacterial activity against 96% of the *Klebsiella* strains we tested. The telocins are agnostic in their killing spectra, showing no regard to the sequence type or capsule type of the bacterial target. The discovery of the telocins opens a prospect to mimic nature to modulate and control populations of *Klebsiella* spp., whether in hospital settings or elsewhere, as has been suggested in work with the klebicin KvarIa (*55*). In addition to their prevalence in *Klebsiella* spp., we detected telomere phages in many important pathogens affecting human health and agriculture including *Shigella*, *Salmonella*, and *Pseudomonas*. The characterization of these and other telomere phages could elucidate the role these enigmatic genetic elements play in the evolution and ecology of bacteria in diverse environments.

## MATERIALS AND METHODS

### Data availability

Complete sequence data are available for *K. quasipneumoniae* FK688 (BioProject: PRJNA717371). The accession for sequence data for phage NAR688 is CP072507.2, and that for phage NAR033 is PQ672043. All other genome and protein accessions are available throughout the text and methods. The mass spectrometry proteomic data have been deposited to the ProteomeXchange Consortium via the PRIDE (*56*) partner repository with the dataset identifier PXD058604.

### Genome sequencing

The FK688 genome was sequenced as described previously (*24*). Genomic DNA (gDNA) from AJ027ø688, AJ292ø688, AJ292ø033, AJ033, AJ218 R1, AJ218 R2, and AJ218 R3 was extracted from overnight cultures using the Wizard Genomic DNA Purification Kit (Promega) as per the manufacturer’s instructions.

For AJ027ø688, AJ292ø688, AJ292ø033, and AJ033, high-molecular-weight gDNA was prepared as Oxford Nanopore sequencing libraries according to the manufacturer’s protocols using a ligation sequencing kit (SQK-LSK109, Oxford Nanopore), preceded by an AMPure (Beckman) XP-based size selection (0.6:1, reagent:sample) and with optional shearing omitted. Barcodes provided in the Native Barcoding Expansion 1-12 (EXP-NBD104, Oxford Nanopore) and 13-24 (EXP-NBD114, Oxford Nanopore) kits were used, and libraries were sequenced with an R9.4.1 flow cell (Oxford Nanopore) on a GridION device (Oxford Nanopore). Read data were basecalled using high-accuracy basecalling in guppy version 5.0.16 (*57*) and demultiplexed using guppy barcoder version 5.0.11 (*57*). Reads were downsampled to provide 500,000,000 base pairs (bp) per sample using filtlong version 0.2.1 (*58*). The mean read lengths of downsampled Oxford Nanopore reads were between 20,000 and 35,000 bp for each sample, providing an estimated 75- to 95-fold coverage of a typical *Klebsiella* genome (5.5 Mb). Matched Illumina reads were prepared on a NovaSeq 6000 platform, with 150-bp paired-end Illumina DNA Prep chemistry, and sequenced across two lanes. Assemblies were generated using Unicycler version 0.4.8 (*59*), using both long- and short-read data and default settings. Annotations were created using Prokka version 1.14.6 (*60*) with the *K. pneumoniae* subsp*. pneumoniae* HS11286 genome (GCA_000240185.2) as a guide through the “proteins” function.

For TelA-resistant AJ218 mutants, extracted DNA was quantified using the Qubit double-stranded DNA Broad Range kit. DNA (23–49 ng) underwent NGS library prep using the Illumina DNA Library prep kit, and libraries were sequenced on the Illumina NovaSeq 6000 system using 2 × 150–bp reads to ~5.5 Mb (~1.5 million reads or ~100× raw coverage per sample). Demultiplexing was performed using bcl2fastq2 version 2.20. All processes were carried out according to manufacturers’ instructions.

### Telomere phage identification and comparison

The TelB phage (NZ_JAGKXJ010000013), TelC phage (NZ_CAJZXW010000028), TelD phage (NZ_CABGLL010000034), and TelE phage (CP067601) were identified by subjecting the NAR688 genome to a BLASTN version 2.9.0 (*61*) analysis against the NCBI nucleotide collections database of all bacterial genomes (June 2022). Telomere phages in non-*Klebsiella* genera were identified through iterative BLASTP version 2.9.0 (*61*) searches of the NAR688 protelomerase amino acid sequence against the NCBI protein database (default settings), excluding *Klebsiella* databases, and assessing the genomes associated with protein hits. Genomes were manually assessed for the presence of hallmark telomere phage genes (protelomerase, SopA, and SopB) as well as canonical phage genes (genes encoding phage virion proteins, terminase proteins, regulation of lysis and lysogeny, etc.). NAR033 was identified through BLASTN version 2.9.0 analysis of the NAR688 genome against the genomes of in-house *Klebsiella* strains (*62*). Telomere phage genomes were aligned and visualized using VIPTree version 3.7 (*45*) and clinker version 1.1.0 (*63*).

### Telocin identification

TelB/ImmB (WP_110096654.1 and WP_110096655.1), TelC/ImmC (WP_225376638.1 and WP_225376636.1), TelD/ImmD (WP_256661714.1 and WP_154905061.1), and TelE/ImmE (UME44985.1 and UME44986.1) were identified from their respective phage genomes by interrogating open-reading frames from the presumptive bacteriocin-immunity locus using BLASTP version 2.9.0 (*61*) and HHPred (*64*). Protein structural predictions were performed using AlphaFold2 via ColabFold version 1.5.2 (*65*), and mechanisms of cytotoxicity of each telocin were predicted by comparison with previously characterized proteins returned from DALI (*66*) searches against the full PDB database. Structural graphics were performed using UCSF ChimeraX version 1.6.1 (*67*).

### Pangenome construction

Genome accessions were obtained from a curated list of publicly available *Klebsiella* genomes (*32*). For the *K. pneumoniae* species complex pangenome, 1309 representative whole-genome sequencing assemblies were retrieved from the collection. For the *K. michiganensis* pangenome, all nonredundant, nonanomalous *K. michiganensis* (*n* = 127), *K. pasteurii* (*n* = 19), and *K. grimontii* (*n* = 64) assemblies were extracted (*n* = 210). Assemblies were annotated using Prokka version 1.14.6 (*60*) and then submitted to Roary version 3.12.0 (*68*) for pangenome construction. In the *K. pneumoniae* dataset, 2885 core genes were identified among a total of 49,317 genes. In the *K. michiganensis* dataset, 3032 core genes were identified among a total of 44,590 genes. An alignment of core genes was used for extracting single-nucleotide polymorphism (SNP) sites from each dataset [SNP-sites version 2.5.1 (*69*)], leaving a total of 491,390 core SNPs for the *K. pneumoniae* species complex dataset and 338,963 core SNPs for the *K. michiganensis* dataset. FastTree version 2.1.10 (*70*) was run on the core SNPs to construct phylogenies for each dataset.

### Screening for telomere phages and telocins in pangenomes

To screen for NAR688-like, TelC phage–like, and TelD phage–like phages among the two pangenomes, BLASTN version 2.9.0 (*61*) was run for all assemblies. To retrieve phage gene hits, a threshold of 90% nucleotide identity over 70% query coverage was applied, with all other thresholds applied as per defaults. Gene presence/absence tables for each phage in each genome were constructed manually from the BLAST outputs and plotted onto phylogenetic trees in iTOL version 6.8.1 (*71*).

To confirm that the predicted NAR688-like bacteriophages identified in the *K. pneumoniae* species complex dataset are extrachromosomal, all assemblies were mapped to the reference chromosome of *K. pneumoniae* subsp*. pneumoniae* MGH 78578 (CP000647.1) to exclude likely chromosomal contigs. BLASTN version 2.9.0 was run on remaining contigs to identify extrachromosomal NAR688-like phages. To assess telocin distribution in each dataset, the amino acid sequences of TelA to TelE were subjected to BLASTP version 2.9.0 (*61*) analysis (default settings) against strains predicted to carry telomere phages.

### Purification of NAR688 virions

To induce the production of NAR688 virions from the *K. quasipneumoniae* FK688 strain, an overnight culture of FK688 was refreshed (1:100) in 800 ml of lysogeny broth (LB; 10 g of tryptone, 5 g of NaCl, and 5 g of yeast extract per 1 liter) and grown (37°C, 200 rpm, 2.75 hours) to the midlog phase of culture. Mitomycin C (0.5 μg/ml; Sigma-Aldrich) was added, and the culture was incubated (37°C, 200 rpm) for 3 hours and then centrifuged (11,000*g*, 30 min, 4°C) to pellet cell debris. The supernatant was filtered using 0.45-μm filter paper, then treated with ribonuclease A (1 μg/ml) and deoxyribonuclease (1 μg/ml), and incubated on ice for 30 min. NaCl was added to a final concentration of 1 M, and the solution was incubated on ice for a further 60 min with gentle stirring. Polyethelyne glycol 8000 (PEG; Sigma-Aldrich) was added to 10% of the final concentration and incubated overnight at 4°C with gentle stirring. PEG-precipitated phages were pelleted by centrifugation (11,000*g*, 20 min, 4°C), the supernatant was discarded, and pellets were resuspended in 12 ml of SM buffer [100 mM NaCl, 8 mM MgSO_4_, and 10 mM tris (pH 7.5)]. An equal volume of chloroform was added, and the solution was vortexed for 30 s to remove PEG and then centrifuged (3000*g*, 15 min, 4°C) to separate the organic and aqueous phases. The aqueous phase (~10 ml) was removed, CsCl was added to a final concentration of 0.5 g/ml, and the suspension was layered onto a discontinuous CsCl gradient (2 ml of 5.6 M CsCl, 1.5 ml of 4 M CsCl, and 1.5 ml of 3.6 M CsCl in SM buffer) in a Beckman SW41 centrifuge tube. Gradients were centrifuged (22,000*g*, 2 hours, 4°C), and a sample of the resulting opaque band suspended in the gradient was collected using a syringe piercing the side of the tube. One milliliter of the sample was layered onto a CsCl floatation gradient (2 ml of 7.2 M CsCl, 3 ml of 5 M CsCl, and 6 ml of 3 M CsCl in SM buffer), and gradients were centrifuged (22,000*g*, 2 hours, 4°C). Phage suspensions were dialyzed in 2 liters of SM buffer overnight, replacing the buffer after the first 8 hours of dialysis. The purified phage solution was stored at 4°C between uses.

### Imaging of NAR688 virions by transmission electron microscopy

Grids for imaging were prepared by placing inverted, freshly glow-discharged (30 mA, 0.23 mbar, 30 s) CF200-Cu carbon support film 200-mesh copper grids (ProSciTech) onto 10 μl of samples of CsCl-purified NAR688 for 3 min. Whatman filter paper (Whatman) was used to blot excess fluid from grids. Samples were stained three times (30, 30, and 60 s) with 2% uranyl acetate in Milli-Q water, with blotting after each round of staining. After air drying, grids were imaged using a JEOL JEM-1400 Plus transmission electron microscope at 80 keV, equipped with a high sensitivity bottom-mount complementary metal-oxide semiconductor “Flash” camera.

NAR688 virion size was measured using DigitalMicrograph Software (DM3) (*72*). The mean head diameter and tail length were calculated from measurements of 10 homogeneous virions in a single micrograph.

### NAR688 lysogen preparation and characterization

To assess the range of *Klebsiella* strains susceptible to NAR688 infection, 4 μl of the CsCl-purified NAR688 preparation was spotted onto bacterial lawn cultures prepared by inoculating 4 ml of molten soft LB agar (10 g of tryptone, 5 g of NaCl, 5 g of yeast extract, and 3.5 g of agar per 1 liter) with 200 μl of bacterial overnight cultures and pouring onto LB agar plates (10 g of tryptone, 5 g of NaCl, 5 g of yeast extract, and 15 g of agar per 1 liter). Four microliters of SM buffer was spotted as a control. Activity was observed after overnight incubation at 37°C. Testing was performed in at least two biological replicates in technical duplicate for each strain.

To prepare *Klebsiella* strains lysogenized by NAR688, bacterial regrowth from NAR688 clearance zones on AJ027 and AJ292 lawns was passaged onto LB agar and then screened for NAR688 lysogeny by colony PCR using NAR688-specific primers (table S3). Positive colonies for each strain were subjected to whole-genome sequencing using long and short reads to confirm the presence of the extrachromosomal phage.

The ability of NAR688 to stably lysogenize AJ027ø688 and AJ292ø688 was assessed by performing successive streak plates onto LB agar over 10 days and then screening for NAR688 within colonies used for each passaging step by colony PCR. Briefly, single colonies were picked from streak plates after each passage and suspended in sterile Milli-Q water. Colony suspensions were used to prepare fresh streak plates, and then the remaining colony suspension was boiled at 95°C for 5 min to kill bacteria and release DNA. Boiled colony suspensions were kept at −20°C. PCR using NAR688-specific primers was performed after the final round of passaging. Passaging was performed in three biological replicates.

To assess the effect of NAR688 lysogeny on bacterial growth, 0.5 McFarland standards of AJ027, AJ027ø688, AJ292, and AJ292ø688 were prepared in sterile 0.8% saline. Cell suspensions were diluted 1:100 in sterile LB and sterile 25% LB, and 200 μl of each suspension was aliquoted into a 96-well plate. The plate was maintained in a hydration chamber, and cell density (optical density at 600 nm) was recorded hourly over 24 hours (200 rpm, amplitude of 3 mm, 37°C) in a Tecan Spark 10 M plate reader. Growth curves were performed in biological triplicate and technical duplicate.

### Proteomic sample preparation

To prepare AJ292 and AJ292ø688 cells for proteomic analysis, overnight cultures of each strain were subcultured 1:100 in 20 ml of LB and grown (200 rpm, 37°C) to the midlog phase of culture. One milliliter of each culture was pelleted (132,000 rpm, 90 s), and pellets were washed with 1× phosphate-buffered saline (pH 7.4; 137 mM NaCl, 2.7 mM KCl, 10 mM Na_2_HPO_4_, and 1.8 mM KH_2_HPO_4_) and then snap frozen in liquid nitrogen. Cell pellets were solubilized in 5% SDS and 100 mM tris-HCl with heating at 95°C for 10 min to denature enzymes. Subsequently, samples were further homogenized, and DNA was sheared using probe ultrasonication; samples were then clarified with centrifugation at 24,000*g* for 10 min. The protein content of the supernatant was measured using the Pierce BCA Protein Assay Kit (cat. no. 23225, Thermo Fisher Scientific) as per the manufacturer’s instructions. Samples were then processed using the S-trap protocol as per the manufacturer’s instructions with equal protein amounts (*73*). Eluted peptides were acidified in 1% trifluoroacetic acid and purified using Stage-tips packed with SDB-RPS (Empore) with 20 μg of binding capacity (*74*), and iRT peptides were spiked into all samples before liquid chromatography–tandem mass spectrometry analysis for monitoring chromatographic performance.

### Mass spectrometry

Samples were analyzed using liquid chromatography–tandem mass spectrometry using a Dionex UltiMate 3000 RSLCnano for peptide separation and analyzed with a Q-Exactive HF hybrid quadrupole-Orbitrap mass spectrometer (Thermo Fisher Scientific) with an Acclaim PepMap RSLC analytical column (75 μm by 50 cm, nanoViper, C18, 2 μm, 100 Å; Thermo Fisher Scientific) and an Acclaim PepMap 100 trap column (100 μm by 2 cm, nanoViper, C18, 5 μm, 100 Å; Thermo Fisher Scientific). Online liquid chromatography was performed using 0.1% formic acid (buffer A) and 80% acetonitrile and 0.1% formic acid (buffer B) with a 120-min linear gradient from 7% to 37.5% (B) used for the separation of peptides before electrospray ionization. The mass spectrometer operated in data-dependent analysis mode with 12 fragmentation spectra per duty cycle. Precursor scans were performed with a resolution of 120,000 (200 *m/z*) from 375 to 1,575 *m/z*, an ion target of 3 × 10^6^, and a maximum injection time of 54 ms. Fragmentation spectra were generated using a normalized collision energy of 27 at a resolution of 30,000, an isolation window of 1.4 *m*/*z*, and a starting mass of 120 *m*/*z* and with only charge states 2 to 5 included in the acquisition. A dynamic exclusion of 15 s was applied to all acquired precursors. The raw data files were analyzed using MaxQuant version 1.6.5.0 (*75*, *76*) with standard parameters against the *Klebsiella* AJ292 reference UniProt proteome (accessed September 2021), NAR688, and common contaminants (*75*). Statistical analysis was performed using LFQ-Analyst version 1.2.6 (*77*) based on the ProteinGroup.txt file with no alteration to set parameters.

### Telocin cloning and purification

Telocin gene products were amplified using the Phusion High Fidelity DNA polymerase system (New England Biolabs) and telocin-specific primers (table S3). For *telA*, amplification was performed using gDNA from FK688 cells as a template. For *telB-E*, amplification was performed from gBlocks (Integrated DNA Technologies), which had been ordered with restriction enzyme cut sites and CG clamps appended to the ends of each gene. PCR products were cloned into pET-23a(+) protein expression vectors (Novagen) (table S3).

Telocin-encoding pET-23a(+) vectors (table S3) were transformed by heat shock into *E. coli* BL21 Star (DE3) cells (Invitrogen) for TelA, TelB, TelC, and TelD and *E. coli* C41 Star (DE3) cells (Lucigen) for TelE. Transformants were grown in overnight cultures in LB and then subcultured in 1 liter of Terrific Broth (12 g of tryptone, 24 g of yeast extract, 4 ml of glycerol, 2.31 g of KH_2_PO_4_, and 12.84 g of K_2_HPO_4_ per 1 liter) [ampicillin (100 μg/ml)] for TelA, TelC, TelD, and TelE or 1 liter of LB [ampicillin (100 μg/ml)] for TelB. Cultures were grown to an optical density at 600 nm of 0.6 to 0.8 and then induced with 0.3 mM isopropyl-β-d-thiogalactopyranoside (Astral). Cultures expressing TelA, TelC, TelD, and TelE were incubated overnight (200 rpm, 18°C). Culture expressing TelB was incubated for 3 hours (200 rpm, 37°C).

Cells were collected by centrifugation (5000*g*, 15 min, 4°C), lysed in lysis buffer [50 mM tris (pH 7.5), 400 mM NaCl, 20 mM imidazole, and 0.5 mM MgCl_2_], and then passed two to five times through a cell press (Avestin Emulsiflex C3). Cell debris was pelleted by centrifugation (27,000*g*, 30 min, 4°C), and His-tagged proteins were purified from the supernatant via nickel affinity chromatography. Proteins were eluted from a 5-ml nickel HisTrap HP column (Cytiva) using a gradient (5 to 50%) of 50 mM tris (pH 7.5), 400 mM NaCl, and 1 M imidazole. Telocins were further purified via size exclusion chromatography using a HiLoad 16/600 Superdex 200-pg column (Cytiva) and equilibrated in gel filtration buffer [25 mM tris (pH 7.5), 150 mM NaCl, and 0.5 mM EDTA]. SDS–polyacrylamide gel electrophoresis (SDS-PAGE) with Coomassie staining was performed to confirm the enrichment of desired proteins in eluted fractions. Fractions were concentrated and spiked with 5% (v/v) glycerol as a cryopreservant and then snap frozen. The concentration of each protein was determined using a Bradford assay (Bio-Rad).

### Telocin killing spectra

To assess the killing spectra of TelA to TelE, purified proteins were normalized to a concentration of ~0.5 μg/ml. Four microliters of each telocin suspension was spotted onto bacterial lawn cultures prepared by inoculating 4 ml of molten soft LB agar with 200 μl of bacterial overnight cultures and pouring onto LB agar. Two microliters of gel filtration buffer was spotted as a control. Antibacterial activity was observed after overnight incubation at 37°C. Testing was performed in at least two biological replicates in technical duplicate for each strain. To confirm the activity of TelD against *Klebsiella* strains AJ006, AJ054, and AJ229, ~20 μg of purified protein was spotted onto soft agar overlays prepared as above, with 10 μl of gel filtration buffer used as a control.

### ImmA cloning, function, and membrane localization

ImmA_HT was amplified from gDNA from FK688 using the Phusion High Fidelity DNA polymerase system (New England Biolabs) and ImmA-specific primers (table S3) to append a His-tag to the N terminus of the protein (ImmA_HT). Empty pCJS223 and pCJS223_ImmA_HT vectors (table S3) were transformed into electrocompetent *K. variicola* AJ292 cells by electroporation.

To assess the membrane localization of ImmA, overnight cultures of *K. variicola* AJ292 + pCJS223 and AJ292 + pCJS223_ImmA_HT were subcultured in 400 ml of LB [ampicillin (100 μg/ml)] and grown (200 rpm, 37°C) to an optical density at 600 nm of 0.5 to 0.6. Cultures were induced with anhydrotetracycline (200 ng/ml) and grown for 3 hours before pelleting by centrifugation (SS34 rotor, 5000*g*, 5 min, 4°C). Before pelleting, samples of culture were collected for use in TelA challenge assays. Membranes were purified from cell pellets as described previously (*78*). Purified membranes were fractionated by sucrose gradient fractionation. Briefly, 400 μl of purified membranes was layered on top of a sucrose gradient (1.9-ml layers of 35, 40, 45, 50, 55, and 60% sucrose-5 mM EDTA solutions, layered from most dense to least dense) in an SW40 tube. Gradients were centrifuged in an ultracentrifuge (34,000 rpm, 19 hours, 4°C) and then fractionated using an ISCO fractionator, with a 70% sucrose solution used as the displacing fluid. Fractions (1 ml) were collected and subjected to SDS-PAGE (12% acrylamide) followed by Coomassie staining and immunoblot analysis. For the immunoblot analysis, an anti-HisTag antibody (R&D Systems) was used to probe for ImmA_HT, an anti-BamC primary antibody (*79*) and anti-Rabbit IgG-peroxidase conjugate secondary antibody (Sigma-Aldrich) were used to probe for the outer-membrane protein BamC, and an anti-PpiD primary antibody (*80*) and anti-Rabbit IgG-peroxidase conjugate secondary antibody (Sigma-Aldrich) were used to probe for the inner-membrane protein, PpiD.

To assess the function of ImmA, 200 μl of AJ292 + pCJS223 and AJ292 + pCJS223_ImmA_HT cultures collected as described previously was added to 4 ml of molten soft LB agar and poured onto LB agar plates. A 10-fold dilution series of TelA with a starting dose of ~3.6 μg/ml was spotted onto plates, with gel filtration buffer used as a control. Plates were incubated overnight at 37°C.

### TelA-resistant mutants

Breakthrough colonies were picked from clearance zones of *K. pneumoniae* strain AJ218 treated with TelA and streaked onto LB agar plates to ensure purity. Single colonies were used to inoculate overnight cultures in LB (37°C), which were then challenged with TelA in further spot testing assays. TelA-resistant mutants R1, R2, and R3 were sequenced as described above. Sequencing reads were trimmed with Trimmomatic (version 0.36) (*81*) and assessed for SNPs relative to the AJ218 wild-type reference genome using Snippy 4.6.0 (*82*).

Wild-type AJ218 *tonB*, *exbB*, and *ompk36* genes were obtained as gBlocks (Integrated DNA technologies) and cloned into a pJP168.Cm expression vector (table S3). Empty vectors and complementation vectors were transformed into electrocompetent AJ218 R1, R2, and R3 strains by electroporation. Overnight cultures of each strain were subcultured 1:50 in 10 ml of LB broth under chloramphenicol (34 μg/ml) selection, grown to an optical density at 600 nm of 0.5 to 0.7, and then induced with anhydrotetracycline (200 ng/ml) for 4 hours. Cultures were used to inoculate soft agar overlays, which were then spotted with a 10-fold dilution series of TelA (starting at ~2 μg) and incubated overnight at 37°C.

### OmpK36 loop chimeras

Empty pJP.168_Cm expression vectors and pJP.168_Cm vectors harboring OmpK36 loop chimera genes generated previously (table S3) (*39*) were transformed into electrocompetent AJ218∆OmpK36 cells by electroporation. Overnight cultures of each strain were subcultured 1:100 in 200 ml of LB in 500-ml flasks with chloramphenicol (34 μg/ml) selection and anhydrotetracycline induction (200 ng/ml) from the time of inoculation. After 7 hours of incubation (200 rpm, 37°C), cultures were used to inoculate soft agar overlays for spot testing assays and the remaining volumes were harvested for membrane purification, as described previously (*78*).

Purified membranes were normalized by total protein concentration in SDS loading buffer and run on 12% SDS-PAGE gels. OmpK36 immunoblots were probed using polyclonal rabbit sera raised against OmpK37 [1:30,000 dilution; (*39*)], which has cross-reactivity with OmpK36. Polyclonal rabbit sera raised against outer membrane lipoprotein BamC [1:60,000 dilution; (*79*)] were used for loading controls. Rabbit antisera [1:20,000 dilution; (*79*)] were used for secondary probing.

### GFP-expressing strains

Constitutively GFP-expressing AJ218 and AJ218∆OmpK36 strains were created following the protocol described previously (*83*) with modifications. A pGRG36 plasmid bearing GFP in the transgene site and a kanamycin resistance marker was transferred to target strains via conjugation with an *E. coli* S17-1 Lambda donor strain at 30°C. Transconjugants were passaged onto LB-Kan-Amp plates and grown overnight (30°C). Colonies were used to inoculate 10 ml of LB with 0.1% arabinose to induce transposon activity and grown overnight (30°C). The resulting culture was diluted and spread on LB agar and then incubated at 42°C overnight to promote plasmid loss. PCR using attTn7_AJ218_F and attTn7_AJ218_R primers (table S3) was performed to detect the chromosomal insertion of the GFP gene. Constitutive expression of GFP was confirmed by imaging colonies under blue light following multiple rounds of passaging.

### Competition experiments

Competition strains were grown in monocultures to the midlog phase, normalized to an optical density at 600 nm of 0.6, and used to inoculate 50 ml of Luria broth in a 5:1 predator:prey ratio by volume. Monocultures of each strain were included as growth controls. Samples of each mixed and monoculture were plated at appropriate dilutions at the time of inoculation (*T* = 0) and at *T* = 3, 6, 9, 12, and 24 hours of growth following inoculation. After incubation, colonies grown on each agar plate were imaged under blue light in an Amersham Imager. Using this technique, colonies lacking GFP (predator strains) appear gray and colonies expressing GFP (prey strains) appear black. Competition experiment was performed in three biological replicates across three independent experiments. Across 30 agar plates (five time points plated in technical duplicate, performed in biological triplicate) obtained for the cross of AJ292ø688 and AJ218, a total of three outlier AJ218 colonies across two agar plates was detected.

## Supplementary Materials

### The PDF file includes:

Figs. S1 to S10 Tables S1 to S3 Legends for data S1 and S2 References
