# Supplementary material for: Telomere bacteriophages are widespread and equip their bacterial hosts with potent interbacterial weapons
Source: Sci Adv. 2025 Apr 30;11(18):eadt1627. doi: 10.1126/sciadv.adt1627 (PMC12042878; doi:10.1126/sciadv.adt1627)
Supplement: Supplementary file 1 — Figs. S1 to S10 Tables S1 to S3 Legends for data S1 and S2 References [file sciadv.adt1627_sm.pdf]

Science Advances
AAAS

Supplementary Materials for

Telomere bacteriophages are widespread and equip their bacterial hosts with potent interbacterial weapons

Sally M. H. Byers *et al.*

Corresponding author: Trevor Lithgow, trevor.lithgow@monash.edu

Sci. Adv. **11**, eadt1627 (2025)
DOI: 10.1126/sciadv.adt1627

The PDF file includes:

Figs. S1 to S10
Tables S1 to S3
Legends for data S1 and S2
References

Other Supplementary Material for this manuscript includes the following:

Data S1 and S2

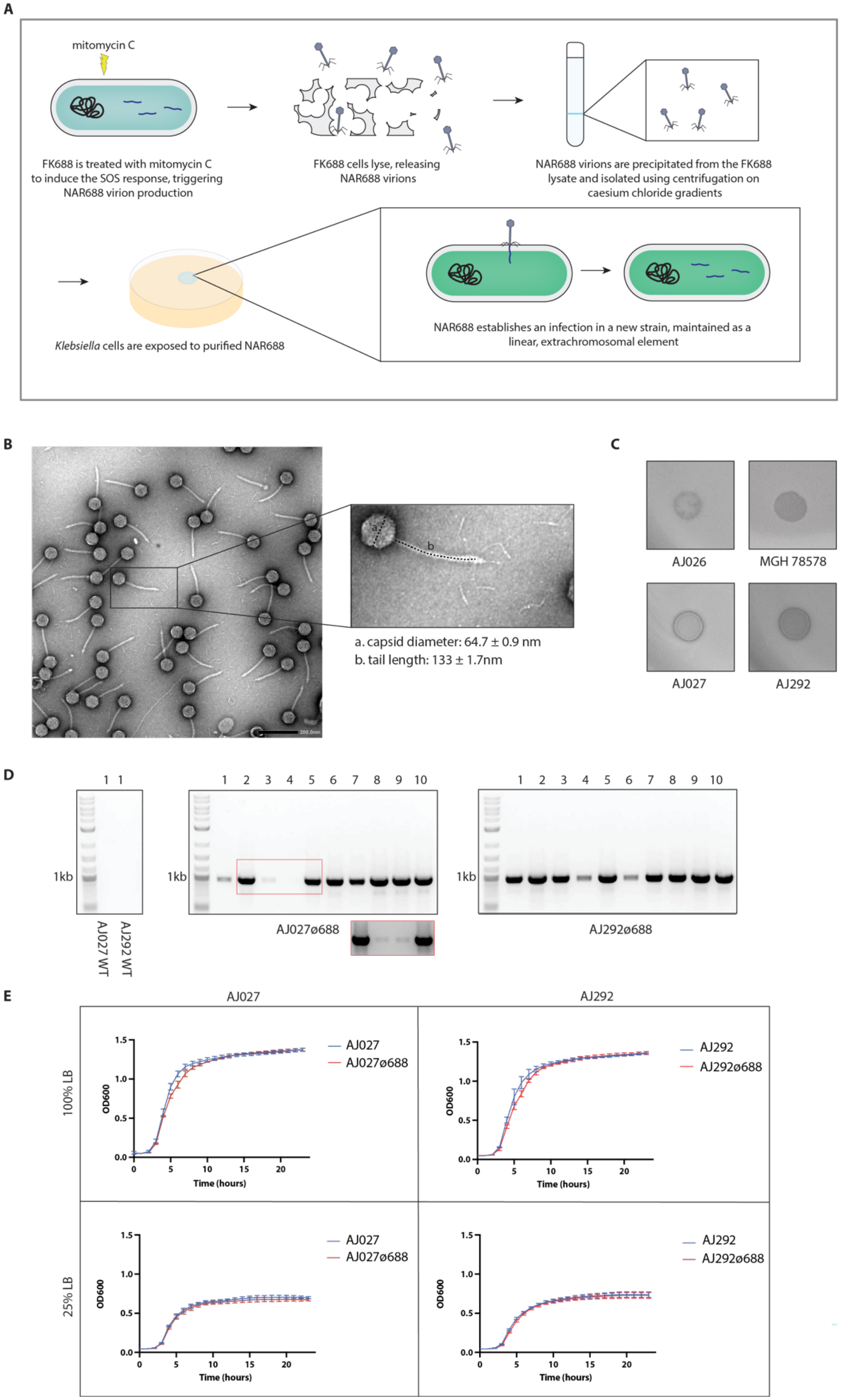

Fig. S1. NAR688 can lysogenise *Klebsiella* strains AJ027 and AJ292. (A) Schematic of preparation of AJ027ø688 and AJ292ø688. NAR688 was induced from FK688 cells by mitomycin C induction. Purified NAR688 was used to infect AJ027 and AJ292 cells, producing AJ027ø688 and AJ292ø688. **(B)** TEM of CsCl-purified NAR688 virions.

Capsid diameter and tail length measurements represented as mean ± SD of ten virion particles. **(C)** Clearance zones of *Klebsiella* AJ026, MGH 78578, AJ027 and AJ292 lawns spotted with purified NAR688. Lytic-like clearance patterns are perceptible on AJ026 and MGH 78578, while AJ027 and AJ292 exhibit bull's eye clearance zones, indicative of phage lysogeny. **(C)** Colony PCR using NAR688-specific primers of AJ027ø688 and AJ292ø688 over 10 successive days of passaging. Numbers indicate day. Red box inset: higher contrast image. **(D)** Growth curves of AJ027, AJ027ø688, AJ292 and AJ292ø688 in 100% and 25% LB. Error bars indicate mean ± SD of three biological replicates performed in technical duplicate.

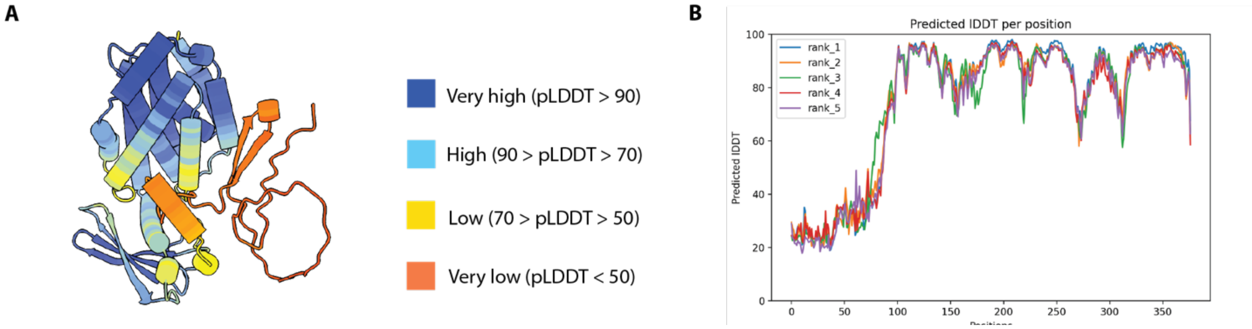

Fig. S2. Telocin A structural prediction. (A) The structure of Telocin A predicted by AlphaFold2, coloured by predicted local distance difference test (pLDDT) scores. Color coding of the scores is shown. **(B)** Per-residue pLDDT scores for the five AlphaFold2-generated models of the Telocin A structure.

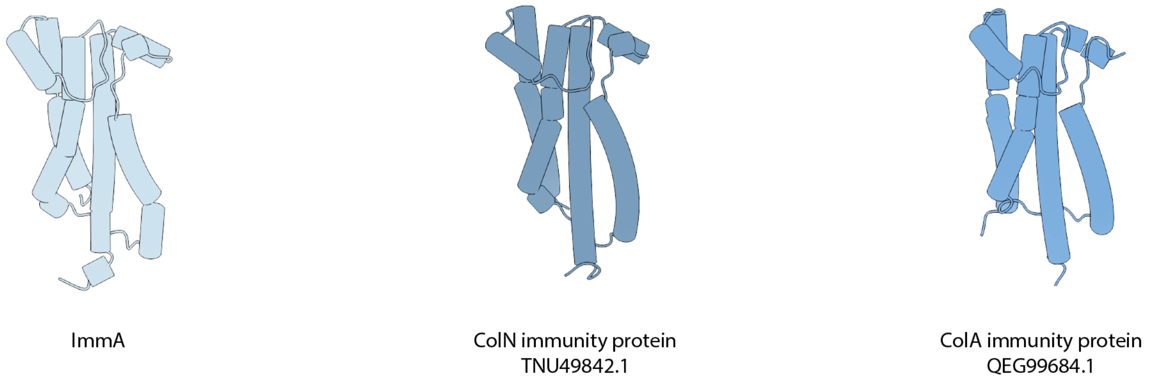

Fig. S3. ImmA, is a homologue of pore-forming bacteriocin immunity proteins. AlphaFold2 structural predictions of ImmA, the immunity proteins of pore forming Colicin N and and the immunity proteins of pore forming Colicin A.

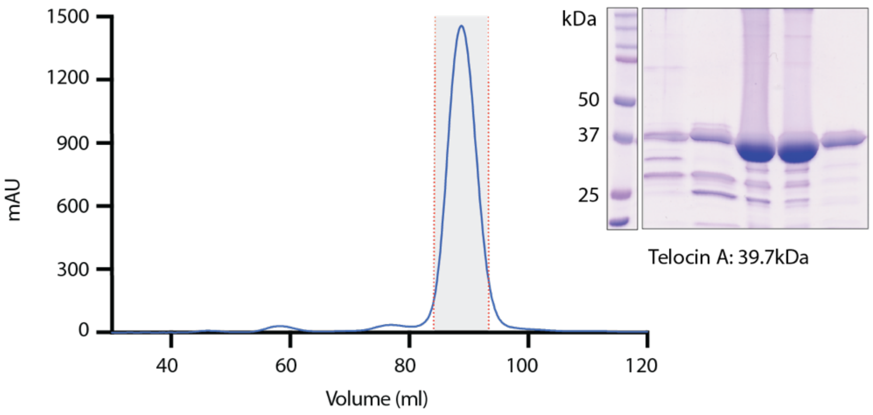

Fig. S4. Purification of Telocin A. Size exclusion chromatogram obtained during the purification of Telocin A. Fractions falling within grey shaded area were pooled and concentrated. This fraction was used to test TelA activity. Inset: Fractions from within the grey-shaded are analyzed by SDS-PAGE and Coomassie blue staining to assess protein purity.

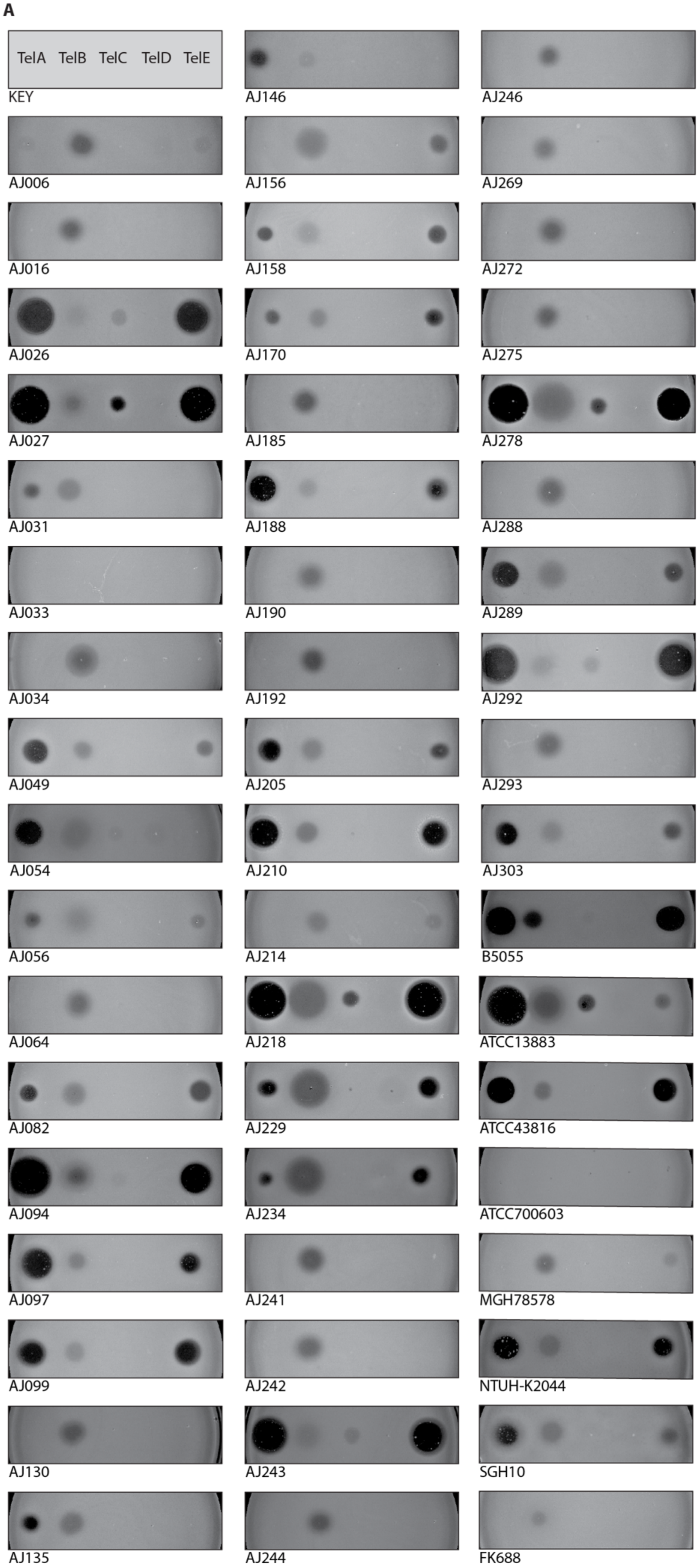

B

		Telocin				
**Strain**	**Capsule type**	**A**	**B**	**C**	**D**	**E**
AJ006	K28	borderline activity	activity		borderline activity	borderline activity
AJ016	K54		activity			
AJ026	K17	activity	borderline activity	activity		activity
AJ027	K81	activity	activity	activity		activity
AJ031	K6	activity	activity			
AJ033	K0					
AJ034	K54		activity			
AJ049	K64	activity	activity			activity
AJ054	K27	activity	activity	activity	borderline activity	
AJ056	K49	activity	activity			activity
AJ064	K54		activity			
AJ082	K11	activity	activity			activity
AJ094	K2	activity	activity	borderline activity		activity
AJ097	K2	activity	activity			activity
AJ099	K2	activity	activity			activity
AJ130	K54		activity			
AJ135	K60	activity	activity			
AJ146	K9	activity	borderline activity			
AJ156	K25		activity			activity
AJ158	K22	activity	activity			activity
AJ170	K38	activity	activity			activity
AJ185	K54		activity			
AJ188	K5	activity	activity			activity
AJ190	K54		activity			
AJ192	K54		activity			
AJ205	K30	activity	activity			activity
AJ210	K2	activity	activity			activity
AJ214	K3		activity			activity
AJ218	K54	activity	activity	activity		activity
AJ229	K26	activity	activity		borderline activity	activity
AJ234	K54	activity	activity			activity
AJ241	K54		activity			
AJ242	K54		activity			
AJ243	K54	activity	activity	activity		activity
AJ244	K54		activity			
AJ246	K54		activity			
AJ269	K54		activity			
AJ272	K54		activity			
AJ275	K54		activity			
AJ278	K2	activity	activity	activity		activity
AJ288	K54		activity			
AJ289	K16	activity	activity			activity
AJ292	K61	activity	borderline activity	activity		activity
AJ293	K54		activity			
AJ303	K21	activity	activity			activity
B5055	K2	activity	activity	activity		activity
ATCC13883	K3	activity	activity	activity		activity
ATCC43816	K2	activity	activity			activity
ATCC700603	K6					
MGH78578	K52		activity			activity
SGH10	K1	activity	activity			activity
NTUH-K2044	K1	activity	activity			activity
FK688	K60		activity			
**Total sensitive strains**		**31**	**51**	**10**	**3**	**30**

activity; borderline activity; no activity (blank cell)

Fig. S5. Killing spectra of the telocins. (A) Spot testing of purified TelA, TelB, TelC, TelD and TelE (approx. 2 μg each) on soft agar overlays freshly inoculated with *Klebsiella* strains and incubated overnight. **(B)** Summary of spot testing results.

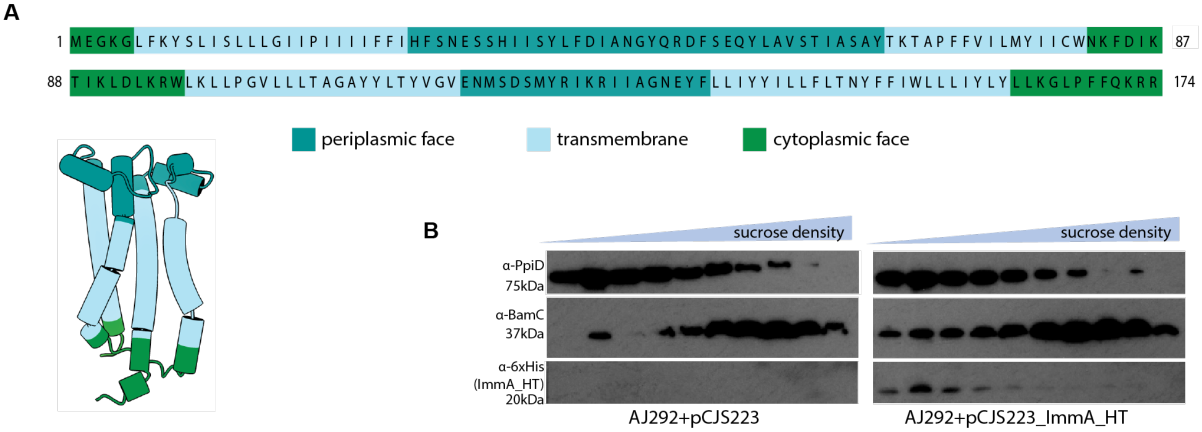

Fig. S6. ImmA localizes to the inner membrane of *Klebsiella* cells. (A) Amino acid sequence and AlphaFold2-generated structural prediction of ImmA, colored by predicted topological features. **(B)** Extracts of AJ292 cells harboring plasmid pCJS223 or pCJS223_ImmA_HT (His-tagged ImmA) were centrifuged on sucrose density gradients which were then fractionated for analysis by SDS-PAGE and immunoblotting. PpiD: inner membrane control. BamC: outer membrane control.

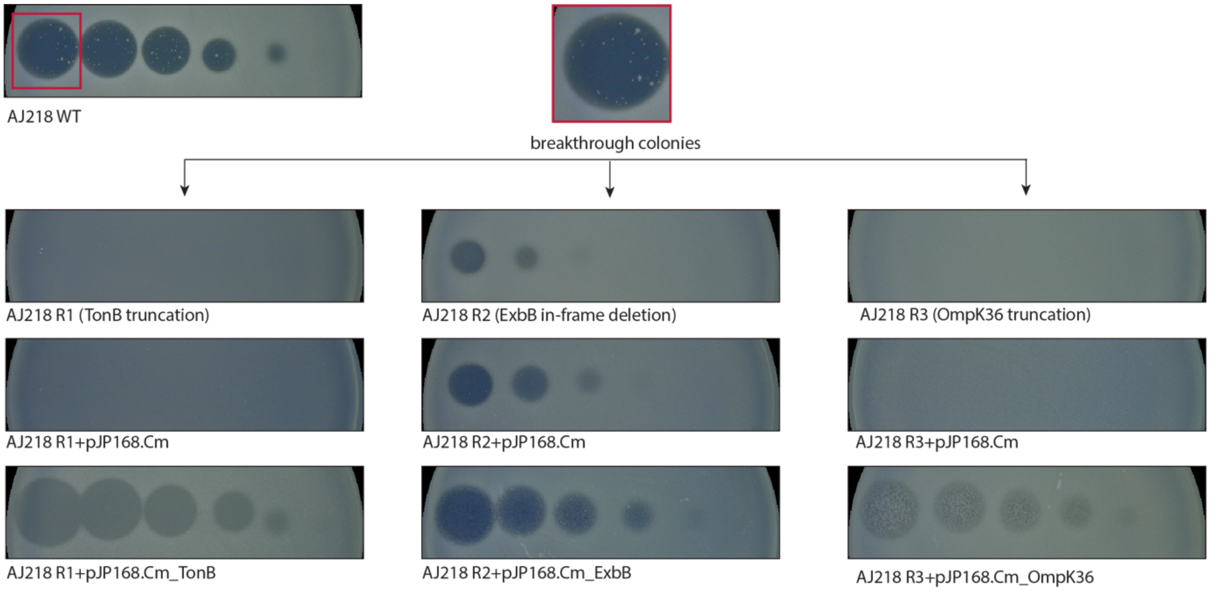

Fig. S7. OmpK36, TonB and ExbB are necessary for sensitivity to Telocin A. (A) Breakthrough colonies R1, R2 and R3 obtained from clearance zones of *K. pneumoniae* strain AJ218 exposed to Telocin A were sub-cultured and shown to be completely (R1, R3) or partially (R2) resistant to Telocin A. Complementation of R1, R2 and R3 with wildtype *tonB, exbB* and *ompK36* genes, respectively, restored sensitivity to Telocin A.

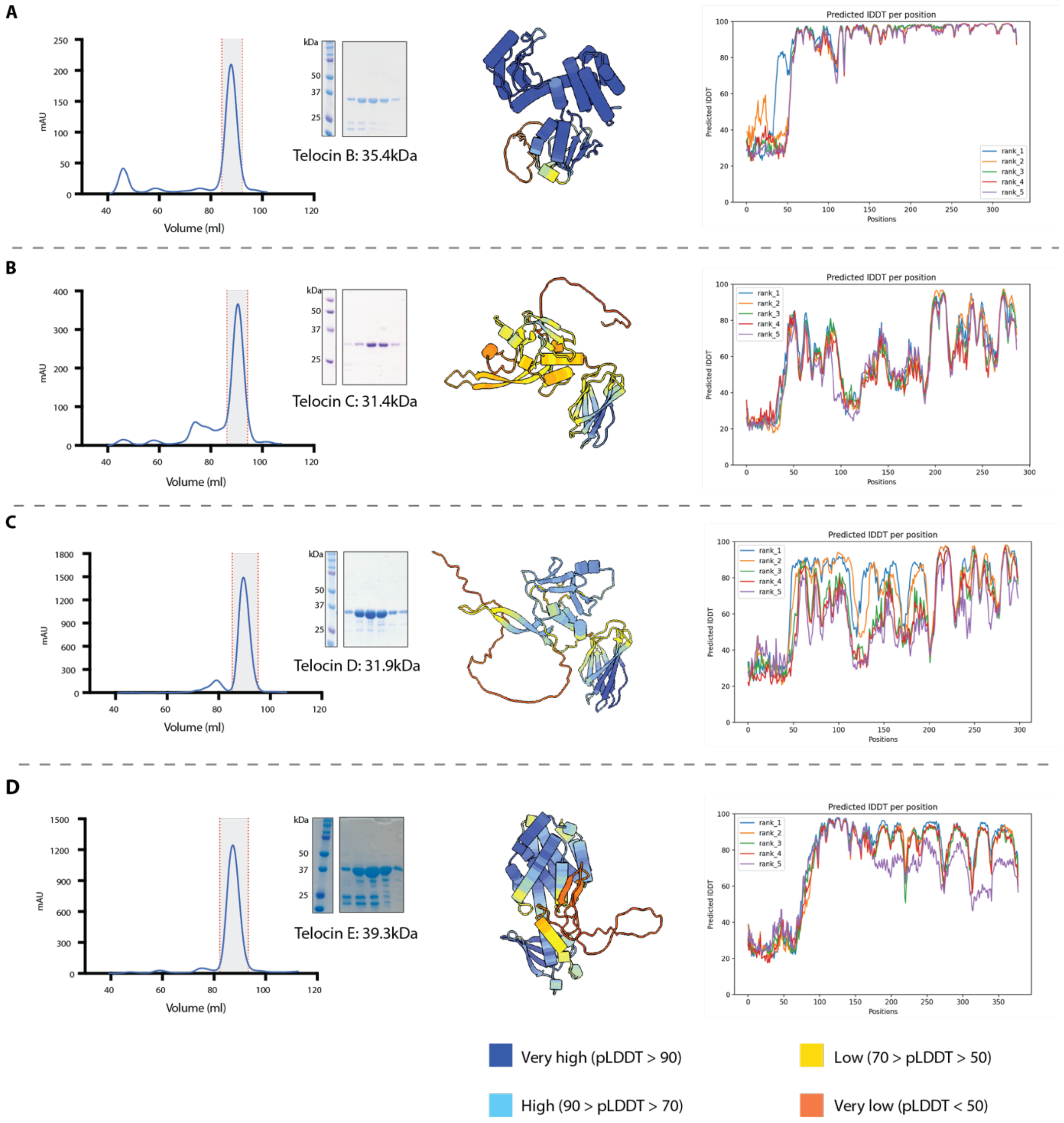

Fig. S8. Purification and structural prediction of telocins B-E. Size exclusion chromatograms obtained during the purification of **(A)** telocin B, **(B)** telocin C, **(C)** telocin D, and **(D)** telocin E, were annotated with grey shading to highlight the fractions which were pooled and concentrated to achieve final telocin preparations. Selected fractions from within grey areas were analyzed by SDS-PAGE and Coomassie blue staining to assess protein purity. INSETS: AlphaFold2-generated predictions of telocin structures, colored by predicted local distance difference test (pLDDT scores), and per-residue pLDDT scores for the five AlphaFold2-generated models of each protein structure.

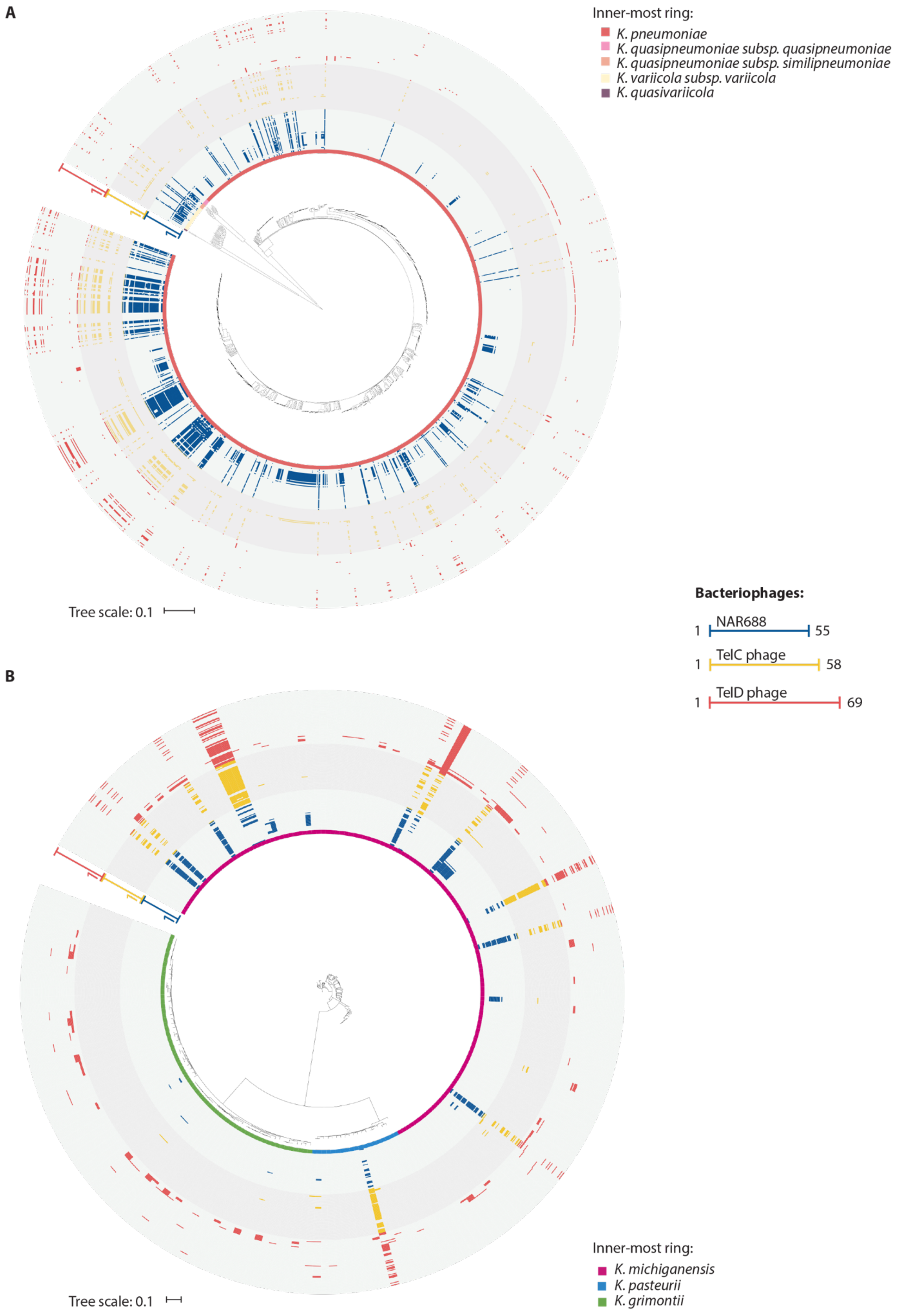
A
Inner-most ring:
K. pneumoniae
K. quasipneumoniae subsp. quasipneumoniae
K. quasipneumoniae subsp. similipneumoniae
K. variicola subsp. variicola
K. quasivariicola
Tree scale: 0.1
Bacteriophages:
1 NAR688 55
1 TelC phage 58
1 TelD phage 69
B
Tree scale: 0.1
Inner-most ring:
K. michiganensis
K. pasteurii
K. grimontii

Fig. S9. Distribution of telomere phage genomes. (A) Phylogenetic tree of genomes (n=1309) included in the *K. pneumoniae* species complex dataset. Species of each genome indicated in the inner-most ring. Presence (≥ 90% identity over 70% gene coverage; coloured tile) or absence (grey tile) of each NAR688, TelC phage and TelD phage gene indicated for each genome. Phage genes are presented in the order in which they appear in their respective genomes. **(B)** Phylogenetic tree of genomes (n=210) included in the *K. michiganensis* dataset. Species of each genome indicated in the inner-most ring. Presence (≥ 90% identity over 70% coverage; colored tile) or absence (grey tile) of each NAR688, TelC phage and TelD phage gene indicated for each genome. Phage genes are presented in the order in which they appear in respective genomes.

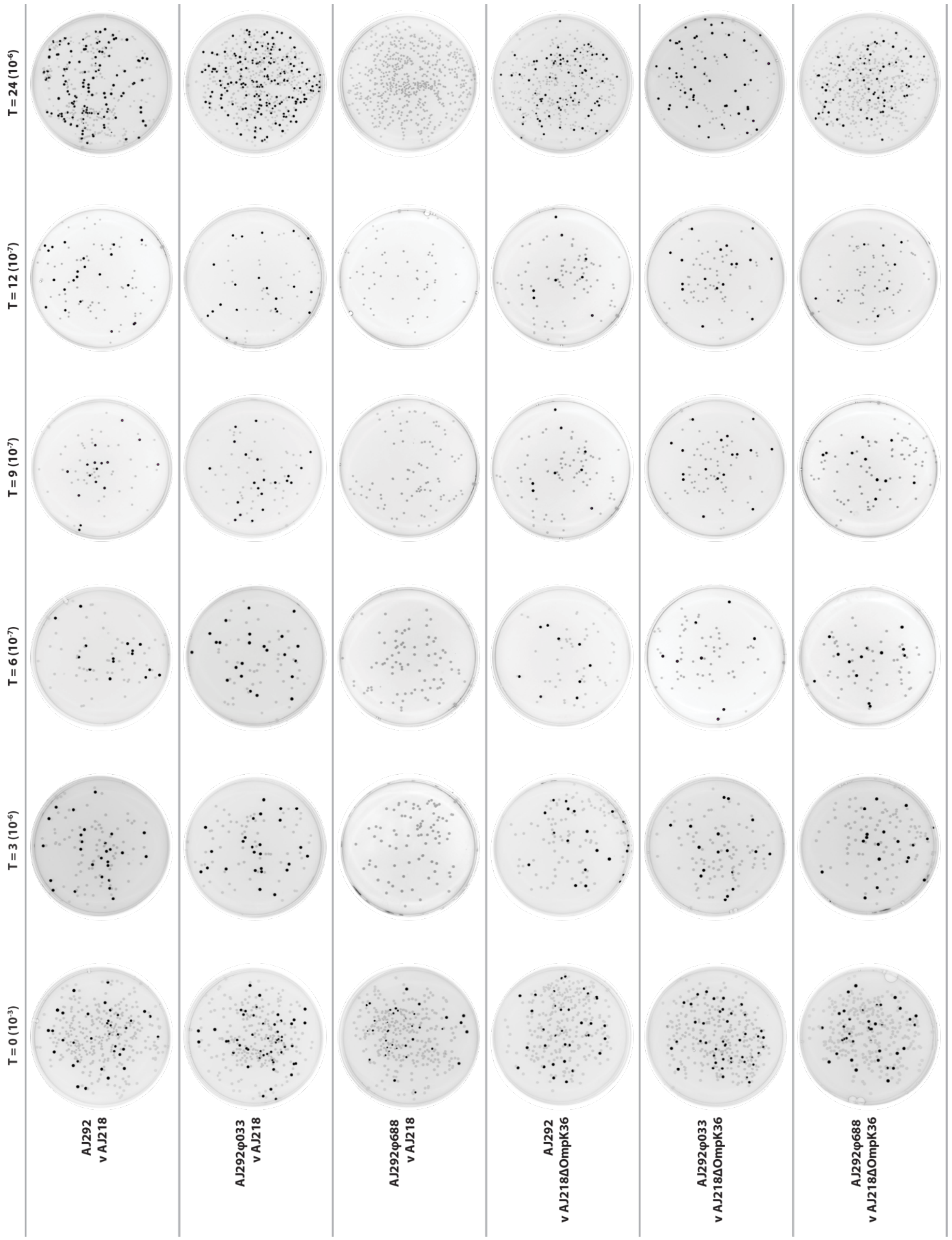

Fig. S10. Full time series for NAR688 lysogen competition experiment. *K. variicola* strains AJ292, AJ292ø033 and AJ292ø688 were co-cultured with GFP-expressing *K. pneumoniae* strains AJ218 and AJ218ΔOmpK36 over a 24-hour period. At the time of inoculation (T = 0) and at T = 3, 6, 9, 12 and 24 hours post-inoculation, samples of each mixed culture were plated onto LB agar plates, then imaged under blue light in an Amersham Imager after overnight incubation. Representative plates from the T = 0, T = 3, T = 6, T = 9, T = 12 and T = 24-hour time points

(dilution factors in brackets) are shown. GFP-positive AJ218 and AJ218ΔOmpK36 colonies appear black, while GFP-negative AJ292, AJ292ø033 and AJ292ø688 colonies appear grey. Experiment performed in three biological replicates across three independent experiments.

Table S1. NAR688 predicted protein annotations

Gene locus tag	Gene product	Predicted protein function
GLDMLFKL_00001	gp1	plasmid-partitioning protein ParA/SopA
GLDMLFKL_00002	gp2	ParB/RepB/Spo0J family plasmid partition protein/SopB
GLDMLFKL_00003	gp3	tail fibre domain-containing protein
GLDMLFKL_00004	gp4	tail protein, carbohydrate binding domain-containing protein, smc-like
GLDMLFKL_00005	gp5	tail assembly protein
GLDMLFKL_00006	gp6	DNA recombinase/translocase domain-containing protein
GLDMLFKL_00007	gp7	C40 family peptidase
GLDMLFKL_00008	gp8	phage minor tail protein L
GLDMLFKL_00009	gp9	phage tail protein
GLDMLFKL_00010	gp10	phage tail tape measure protein
GLDMLFKL_00011	gp11	phage tail protein
GLDMLFKL_00012	gp12	major tail shaft subunit
GLDMLFKL_00013	gp13	HK97 gp10 family phage protein
GLDMLFKL_00014	gp14	tail terminator
GLDMLFKL_00015	gp15	head-tail adaptor protein
GLDMLFKL_00016	gp16	phage gp6-like head-tail connector protein
GLDMLFKL_00017	gp17	Gp7 phage protein
GLDMLFKL_00018	gp18	phage major capsid protein
GLDMLFKL_00019	gp19	S49 family peptidase
GLDMLFKL_00020	gp20	phage portal protein
GLDMLFKL_00021	gp21	terminase large subunit
GLDMLFKL_00022	gp22	P27 family phage terminase small subunit
GLDMLFKL_00023	gp23	hypothetical protein
GLDMLFKL_00024	gp24	hypothetical protein
GLDMLFKL_00025	gp25	hypothetical protein
GLDMLFKL_00026	gp26	hypothetical protein
GLDMLFKL_00027	gp27	nucleoside-2-deoxyribosyltransferase
GLDMLFKL_00028	gp28	hypothetical protein
GLDMLFKL_00029	gp29	hypothetical protein
GLDMLFKL_00030	gp30	hypothetical protein
GLDMLFKL_00031	gp31	hypothetical protein
GLDMLFKL_00032	gp32	site-specific DNA-methyltransferase
GLDMLFKL_00033	gp33	hypothetical protein
GLDMLFKL_00034	gp34	hypothetical protein
GLDMLFKL_00035	gp35	type II toxin-antitoxin system RelE/ParE family toxin
GLDMLFKL_00036	gp36	helix-turn-helix transcriptional regulator
GLDMLFKL_00037	gp37	hypothetical protein
GLDMLFKL_00038	gp38	hypothetical protein
GLDMLFKL_00039	gp39	hypothetical protein
GLDMLFKL_00040	gp40	hypothetical protein

GLDMLFKL_00041	gp41	hypothetical protein
GLDMLFKL_00042	gp42	hypothetical protein
GLDMLFKL_00043	gp43	DNA polymerase III PolC-type
GLDMLFKL_00044	gp44	Q-like phage antitermination protein
GLDMLFKL_00045	gp45	Cro-like lytic repressor
GLDMLFKL_00046	gp46	cB-like prophage repressor protein
GLDMLFKL_00047	gp47	origin of replication binding family protein, RepA
GLDMLFKL_00048	gp48	hypothetical protein
GLDMLFKL_00049	gp49	hypothetical protein
GLDMLFKL_00050	gp50	ImmA
GLDMLFKL_00051	gp51	Telocin A
GLDMLFKL_00052	gp52	host cell division inhibitor Icd-like protein
GLDMLFKL_00053	gp53	AntA-like protein
GLDMLFKL_00054	gp54	AntB-like protein
GLDMLFKL_00055	gp55	Protelomerase

Table S2. Test strains for phage NAR688 and telocin sensitivity

Strain	Capsule type	Reference	Source
AJ006	K28	(*2, 62*)	Dr. Adam Jenney, Alfred Hospital
AJ016	K54	(*62*)	Dr. Adam Jenney, Alfred Hospital
AJ026	K17	(*2, 62*)	Dr. Adam Jenney, Alfred Hospital
AJ027	K81	(*2, 62*)	Dr. Adam Jenney, Alfred Hospital
AJ031	K6	(*2, 62*)	Dr. Adam Jenney, Alfred Hospital
AJ033	K0	(*2, 62*)	Dr. Adam Jenney, Alfred Hospital
AJ034	K54	(*2, 62*)	Dr. Adam Jenney, Alfred Hospital
AJ049	K64	(*2, 62*)	Dr. Adam Jenney, Alfred Hospital
AJ054	K27	(*2, 62*)	Dr. Adam Jenney, Alfred Hospital
AJ056	K49	(*2, 62*)	Dr. Adam Jenney, Alfred Hospital
AJ064	K54	(*2, 62*)	Dr. Adam Jenney, Alfred Hospital
AJ082	K11	(*2, 62*)	Dr. Adam Jenney, Alfred Hospital
AJ094	K2	(*2, 62*)	Dr. Adam Jenney, Alfred Hospital
AJ097	K2	(*2, 62*)	Dr. Adam Jenney, Alfred Hospital
AJ099	K2	(*2, 62*)	Dr. Adam Jenney, Alfred Hospital
AJ130	K54	(*62*)	Dr. Adam Jenney, Alfred Hospital
AJ135	K60	(*2, 62*)	Dr. Adam Jenney, Alfred Hospital
AJ146	K9	(*2, 62*)	Dr. Adam Jenney, Alfred Hospital
AJ156	K25	(*2, 62*)	Dr. Adam Jenney, Alfred Hospital
AJ158	K22	(*2, 62*)	Dr. Adam Jenney, Alfred Hospital
AJ170	K38	(*2, 62*)	Dr. Adam Jenney, Alfred Hospital
AJ185	K54	(*62*)	Dr. Adam Jenney, Alfred Hospital
AJ188	K5	(*2, 62*)	Dr. Adam Jenney, Alfred Hospital
AJ190	K54	(*62*)	Dr. Adam Jenney, Alfred Hospital
AJ192	K54	(*62*)	Dr. Adam Jenney, Alfred Hospital
AJ205	K30	(*2, 62*)	Dr. Adam Jenney, Alfred Hospital
AJ210	K2	(*2, 62*)	Dr. Adam Jenney, Alfred Hospital
AJ214	K3	(*2, 62*)	Dr. Adam Jenney, Alfred Hospital
AJ218	K54	(*2, 62*)	Dr. Adam Jenney, Alfred Hospital
AJ229	K26	(*2, 62*)	Dr. Adam Jenney, Alfred Hospital
AJ234	K54	(*62*)	Dr. Adam Jenney, Alfred Hospital
AJ241	K54	(*62*)	Dr. Adam Jenney, Alfred Hospital
AJ242	K54	(*62*)	Dr. Adam Jenney, Alfred Hospital
AJ243	K54	(*62*)	Dr. Adam Jenney, Alfred Hospital
AJ244	K54	(*62*)	Dr. Adam Jenney, Alfred Hospital
AJ246	K54	(*62*)	Dr. Adam Jenney, Alfred Hospital
AJ269	K54	(*62*)	Dr. Adam Jenney, Alfred Hospital
AJ272	K54	(*62*)	Dr. Adam Jenney, Alfred Hospital
AJ275	K54	(*62*)	Dr. Adam Jenney, Alfred Hospital
AJ278	K2	(*2, 62*)	Dr. Adam Jenney, Alfred Hospital
AJ288	K54	(*62*)	Dr. Adam Jenney, Alfred Hospital
AJ289	K16	(*2, 62*)	Dr. Adam Jenney, Alfred Hospital
AJ292	K61	(*2, 62*)	Dr. Adam Jenney, Alfred Hospital
AJ293	K54	(*62*)	Dr. Adam Jenney, Alfred Hospital
AJ303	K21	(*2, 62*)	Dr. Adam Jenney, Alfred Hospital
B5055	K2	Statens Serum Institut, Denmark	Prof. Richard Strugnell, University of Melbourne
ATCC 1388	K3	ATCC 13883™	American Type Culture Collection
ATCC 43816	K2	ATCC 43816™	American Type Culture Collection

ATCC 700603	K6	ATCC 700603™	American Type Culture Collection
MGH 78578/ ATCC 700721	K52	MGH 78578/ ATCC 700721™	American Type Culture Collection
SGH10	K1	(*84, 85*)	Dr. Francesca Short, Monash University
NTUH-K2044	K1	(*86*)	Dr. Francesca Short, Monash University
FK688	K60	(*24*)	Prof. Tieli Zhou, The First Affiliated Hospital of Wenzhou Medical University, China

Table S3. Primers, plasmids and *Escherichia coli* strains used in this study

Primers			
**Primer Name**	**Primer Sequence (underline: restriction enzyme cut sites)**	**Cloning restriction enzymes**	**Description**
NAR688_F<br>NAR688_R	5'-CTCGTTCACAAGCGAATTG-3'<br>5'-GTAACTCAAAATCAGGTGGTGG-3'	N/A<br>N/A	Detection of NAR688 in lysogenic strains
TelA_F<br>TelA_R	5'-GCGCGCCATATGCCTGAAGAAACATTGACTGTC-3'<br>5'-CGCCGCTCGAGTTTAAATAAGCCAAGCACTGC-3'	NdeI<br>XhoI	Amplification of *telA* gene from NAR688 genome
ImmA_F<br>ImmA_R	5'- GGCGGCCCATGGGTGAAGGGAAGGGGCTGTTCA -3'<br>5’- CGCGCGGGATCCGTAAAGCGGAGGTTATCTCCGC -3’	NcoI<br>BamHI	Amplification of *immA* gene from NAR688 genome
TelB_F<br>TelB_R	5’-GCGCGCCATATGCCAGAAGAAACCATGACTGTTA-3’<br>5’-CGCCGCTCGAGTGGTAATTTACCAGCGTCCAAATCAC-3'	NdeI<br>XhoI	Amplification of *telB* gene from gBlock
TelC_F<br>TelC_R	5’-GCGCGCGCTAGCGCTTATGAAGAAACTATGACGTC-3’<br>5’-CGCCGCTCGAGATAGGTAATTTTTATATTTTGACCGATGG-3'	NheI<br>XhoI	Amplification of *telC* gene from gBlock
TelD_F<br>TelD_R	5’-GCGCGCGCTAGCGGTGGATTTAACTACGGTGGTC-3’<br>5’-CGCCGCTCGAGATAGTTGACTCTTATGTTTTGGCC-3’	NheI<br>XhoI	Amplification of *telD* gene from gBlock
TelE_F<br>TelE_R	5’-GCGCGCCATATGCCTGAAGATTCAATGACAG-3’<br>5’-GGCCGCTCGAGTTTGAATAAATCGGCCACGGC-3’	NdeI<br>XhoI	Amplification of *telE* gene from gBlock
TonB_F<br>TonB_R	5'-GCGCGCGGATCCATGAGCGCAATG-3'<br>5'-GCCGCCAAGCTTTCAGTTAATCTCGA-3’	BamHI<br>HindIII	Amplification of *tonB* gene from gBlock
ExbB_F<br>ExbB_R	5'-GCGCGCGGATCCATGGGTAATAATTTG-3<br>5'-GCCGCCAAGCTTTCAACCTAC-3'	BamHI<br>HindIII	Amplification of *exbB* gene from gBlock
pJP_F<br>pJP_R	5'-CCTAATTTTTGTTGACACTCTATCATTG-3'<br>5'-GCCAGGCAAATTCTGTTTTATCAGACCG-3'	N/A<br>N/A	Sequencing pJP168.Cm vectors containing complementation genes
T7_promoter<br>T7_terminator	5'-AATACGACTCACTATAG-3’<br>5’-GCTAGTTATTGCTCAGCGG-3’	N/A<br>N/A	Sequencing pET-23a(+) vectors containing Telocin genes
VVT115<br>VVT207	5'-GATTATGCGGCCGTGTACAA-3'<br>5'-CGATTCCGACCTCATTAAGCAGCTCTAATG-3'	N/A<br>N/A	Sequencing pCJS223 vector containing *immA*_HT gene
attTn7_AJ218_F<br>attTn7_AJ218_R	5’-TAACATGCACATCATTGAGAT-3’<br>5’-GTGGTACGCATAACGTTC-3’	N/A<br>N/A	Confirmation of chromosomal insertion of GFP gene

Plasmids		
**Plasmid**	**Description**	**Source**
pET-23a(+)	Isopropyl ß-D-1-thiogalactopyranoside (IPTG)-inducible protein expression plasmid. Ampicillin resistance marker.	Novagen
pET-23a(+)_*telA*	Telocin A cloning, expression	This study
pET-23a(+)_*telB*	Telocin B cloning, expression	This study
pET-23a(+)_*telC*	Telocin C cloning, expression	This study

pET-23a(+)_*telD*	Telocin D cloning, expression	This study
pET-23a(+)_*telE*	Telocin E cloning, expression	This study
pCJS223	pETDuet-1 (Novagen) derivative with IPTG-inducibility changed to ATC inducibility	Dr. Christopher Stubenrauch & Dr. Von Torres, Monash University
pCJS223_*immA*_HT	ImmA_HT expression in *Klebsiella* cells	This study
pJP168.Cm	Anhydrotetracycline (ATC)-inducible protein expression plasmid derived from pJP168. Chloramphenicol resistance marker.	(*38*)
pJP168.Cm_*tonB*	*tonB* expression in *Klebsiella* cells	This study
pJP168.Cm_*exbB*	*exbB* expression in *Klebsiella* cells	This study
pJP168.Cm_*ompK36*	*ompK36* expression in *Klebsiella* cells	(*38*)
pJP168.Cm_*ompK36-[L1, L2, L4-L8]*	*ompK36 loop chimera* expression in *Klebsiella* cells	(*39*)
	***E. coli* Strains**	
**Strain**	**Purpose**	**Source**
E. coli BL21 Star™ (DE3)	Expression of Telocins A, B, C and D for purification	Invitrogen
E. coli C41 Star DE3	Expression of Telocin E for purification	Lucigen
	**Antibodies**	
**Antibody**	**Source**	**Identifier**
αBamC	(*79*)	N/A
αPpiD	(*80*)	N/A
αRabbit IgG-peroxidase	Sigma	Cat#A6154
αHis Tag	R&D Systems	Cat#MAB050R
Anti-OmpK37 (porin)	(*39*)	N/A

Data S1. (separate file)

K. pneumoniae species complex dataset genome accessions, predicted telomere phages and predicted telocins.

Data S2. (separate file)

K. michiganensis dataset genome accessions, predicted telomere phages and predicted telocins.

REFERENCES AND NOTES

1. S. Brisse, V. Passet, P. A. D. Grimont, Description of *Klebsiella quasipneumoniae* sp. nov., isolated from human infections, with two subspecies, *Klebsiella quasipneumoniae* subsp. *quasipneumoniae* subsp. nov. and *Klebsiella quasipneumoniae* subsp. *similipneumoniae* subsp. nov., and demonstration that *Klebsiella singaporensis* is a junior heterotypic synonym of *Klebsiella variicola*. *Int. J. Syst. Evol. Microbiol.* **64**, 3146–3152 (2014).

2. K. E. Holt, H. Wertheim, R. N. Zadoks, S. Baker, C. A. Whitehouse, D. Dance, A. Jenney, T. R. Connor, L. Y. Hsu, J. Severin, S. Brisse, H. Cao, J. Wilksch, C. Gorrie, M. B. Schultz, D. J. Edwards, K. V. Nguyen, T. V. Nguyen, T. T. Dao, M. Mensink, V. L. Minh, N. T. Nhu, C. Schultsz, K. Kuntaman, P. N. Newton, C. E. Moore, R. A. Strugnell, N. R. Thomson, Genomic analysis of diversity, population structure, virulence, and antimicrobial resistance in *Klebsiella pneumoniae*, an urgent threat to public health. *Proc. Natl. Acad. Sci. U.S.A.* **112**, E3574–3581 (2015).

3. C. Rodrigues, V. Passet, A. Rakotondrasoa, S. Brisse, Identification of *Klebsiella pneumoniae*, *Klebsiella quasipneumoniae*, *Klebsiella variicola* and related phylogroups by MALDI-TOF mass spectrometry. *Front. Microbiol.* **9**, 3000 (2018).

4. Y. Schukken, M. Chuff, P. Moroni, A. Gurjar, C. Santisteban, F. Welcome, R. Zadoks, The "other" Gram-negative bacteria in mastitis: *Klebsiella*, *Serratia*, and more. *Vet. Clin. North Am. Food Anim. Pract.* **28**, 239–256 (2012).

5. B. Brooks, B. A. Firek, C. S. Miller, I. Sharon, B. C. Thomas, R. Baker, M. J. Morowitz, J. F. Banfield, Microbes in the neonatal intensive care unit resemble those found in the gut of premature infants. *Microbiome* **2**, 1 (2014).

6. G. Wareth, H. Neubauer, The Animal-foods-environment interface of *Klebsiella pneumoniae* in Germany: An observational study on pathogenicity, resistance development and the current situation. *Vet. Res.* **52**, 16 (2021).

7. E. Jurkevitch, Riding the Trojan horse: Combating pest insects with their own symbionts. *J. Microbial. Biotechnol.* **4**, 620–627 (2011).

8. A. B. Hadapad, C. S. Prabhakar, S. C. Chandekar, J. Tripathi, R. S. Hire, Diversity of bacterial communities in the midgut of *Bactrocera cucurbitae* (Diptera: Tephritidae) populations and their potential use as attractants. *Pest Manag. Sci.* **72**, 1222–1230 (2016).

9. M. F. Raza, Z. Yao, S. Bai, Z. Cai, H. Zhang, Tephritidae fruit fly gut microbiome diversity, function and potential for applications. *Bull. Entomol. Res.* **110**, 423–437 (2020).

10. A. A. Pinto-Tomás, M. A. Anderson, G. Suen, D. M. Stevenson, F. S. Chu, W. W. Cleland, P. J. Weimer, C. R. Currie, Symbiotic nitrogen fixation in the fungus gardens of leaf-cutter ants. *Science* **326**, 1120–1123 (2009).

11. F. O. Aylward, C. R. Currie, G. Suen, The evolutionary innovation of nutritional symbioses in leaf-cutter ants. *Insects* **3**, 41–61 (2012).

12. K. L. Wyres, K. E. Holt, *Klebsiella pneumoniae* as a key trafficker of drug resistance genes from environmental to clinically important bacteria. *Curr. Opin. Microbiol.* **45**, 131–139 (2018).

13. D. M. P. De Oliveira, B. M. Forde, T. J. Kidd, P. N. A. Harris, M. A. Schembri, S. A. Beatson, D. L. Paterson, M. J. Walker, Antimicrobial resistance in ESKAPE pathogens. *Clin. Microbiol. Rev.* **33**, (2020).

14. Antimicrobial Resistance Collaborators, Global burden of bacterial antimicrobial resistance in 2019: A systematic analysis. *Lancet* **399**, 629–655 (2022).

15. C. M. Marr, T. A. Russo, Hypervirulent *Klebsiella pneumoniae*: A new public health threat. *Expert Rev. Anti Infect. Ther.* **17**, 71–73 (2019).

16. T. A. Russo, C. M. Marr, Hypervirulent *Klebsiella pneumoniae*. *Clin. Microbiol. Rev.* **32**, 10.1128/cmr.00001-19 (2019).

17. M. M. C. Lam, K. L. Wyres, R. R. Wick, L. M. Judd, A. Fostervold, K. E. Holt, I. H. Löhr, Convergence of virulence and MDR in a single plasmid vector in MDR *Klebsiella pneumoniae* ST15. *J. Antimicrob. Chemother.* **74**, 1218–1222 (2019).

18. Y. Chen, K. Marimuthu, J. Teo, I. Venkatachalam, B. P. Z. Cherng, L. De Wang, S. R. S. Prakki, W. Xu, Y. H. Tan, L. C. Nguyen, T. H. Koh, O. T. Ng, Y.-H. Gan, Acquisition of plasmid with carbapenem-resistance gene bla_{KPC2} in hypervirulent *Klebsiella pneumoniae*, Singapore. *Emerg. Infect. Dis.* **26**, 549–559 (2020).

19. J. Hawkey, H. Cottingham, A. Tokolyi, R. R. Wick, L. M. Judd, L. Cerdeira, D. de Oliveira Garcia, K. L. Wyres, K. E. Holt, Linear plasmids in *Klebsiella* and other *Enterobacteriaceae*. *Microb. Genom.* **8**, 000807 (2022).

20. T.-L. Lin, C.-Z. Lee, P.-F. Hsieh, S.-F. Tsai, J.-T. Wang, Characterization of integrative and conjugative element ICE*Kp1*-associated genomic heterogeneity in a *Klebsiella pneumoniae* strain isolated from a primary liver abscess. *J. Bacteriol.* **190**, 515–526 (2008).

21. M. M. C. Lam, R. R. Wick, K. L. Wyres, C. L. Gorrie, L. M. Judd, A. W. J. Jenney, S. Brisse, K. E. Holt, Genetic diversity, mobilisation and spread of the yersiniabactin-encoding mobile element ICE*Kp* in *Klebsiella pneumoniae* populations. *Microb. Genom.* **4**, e000196 (2018).

22. J. Shen, J. Zhou, Y. Xu, Z. Xiu, Prophages contribute to genome plasticity of *Klebsiella pneumoniae* and may involve the chromosomal integration of ARGs in CG258. *Genomics* **112**, 998–1010 (2020).

23. J. A. M. de Sousa, A. Buffet, M. Haudiquet, E. P. C. Rocha, O. Rendueles, Modular prophage interactions driven by capsule serotype select for capsule loss under phage predation. *ISME J.* **14**, 2980–2996 (2020).

24. N. C. Rosas, J. Wilksch, J. Barber, J. Li, Y. Wang, Z. Sun, A. Rocker, C. T. Webb, L. Perlaza-Jiménez, C. J. Stubenrauch, V. Dhanasekaran, J. Song, G. Taiaroa, M. Davies, R. A. Strugnell, Q. Bao, T. Zhou, M. J. McDonald, T. Lithgow, The evolutionary mechanism of non-carbapenemase carbapenem-resistant phenotypes in *Klebsiella* spp. *eLife* **12**, e83107 (2023).

25. P. S. Liew, T. H. Tan, Y. C. Wong, E. U. H. Sim, C. W. Lee, K. Narayanan, A self-replicating linear DNA. *ACS Synth. Biol.* **9**, 804–813 (2020).

26. N. V. Ravin, Replication and maintenance of linear phage-plasmid N15. *Microbiol. Spectr.* **3**, PLAS-0032-2014 (2015).

27. V. N. Rybchin, A. N. Svarchevsky, The plasmid prophage N15: A linear DNA with covalently closed ends. *Mol. Microbiol.* **33**, 895–903 (1999).

28. N. V. Ravin, N15: The linear phage-plasmid. *Plasmid* **65**, 102–109 (2011).

29. V. K. Ravin, M. G. Shulga, Evidence for extrachromosomal location of prophage N15. *Virology* **40**, 800–807 (1970).

30. S. R. Casjens, E. B. Gilcrease, W. M. Huang, K. L. Bunny, M. L. Pedulla, M. E. Ford, J. M. Houtz, G. F. Hatfull, R. W. Hendrix, The pKO2 linear plasmid prophage of *Klebsiella oxytoca*. *J. Bacteriol.* **186**, 1818–1832 (2004).

31. S. Hertwig, I. Klein, R. Lurz, E. Lanka, B. Appel, PY54, a linear plasmid prophage of *Yersinia enterocolitica* with covalently closed ends. *Mol. Microbiol.* **48**, 989–1003 (2003).

32. M. M. C. Lam, R. R. Wick, S. C. Watts, L. T. Cerdeira, K. L. Wyres, K. E. Holt, A genomic surveillance framework and genotyping tool for *Klebsiella pneumoniae* and its related species complex. *Nat. Commun.* **12**, 4188 (2021).

33. T. Y. Thung, M. E. White, W. Dai, J. J. Wilksch, R. S. Bamert, A. Rocker, C. J. Stubenrauch, D. Williams, C. Huang, R. Schittelhelm, J. J. Barr, E. Jameson, S. McGowan, Y. Zhang, J. Wang, R. A. Dunstan, T. Lithgow, Component parts of bacteriophage virions accurately defined by a machine-learning approach built on evolutionary features. *mSystems* **6**, e0024221 (2021).

34. E. Cascales, S. K. Buchanan, D. Duche, C. Kleanthous, R. Lloubes, K. Postle, M. Riley, S. Slatin, D. Cavard, Colicin biology. *Microbiol. Mol. Biol. Rev.* **71**, 158–229 (2007).

35. A. Harms, D. E. Brodersen, N. Mitarai, K. Gerdes, Toxins, targets, and triggers: An overview of toxin-antitoxin biology. *Mol. Cell* **70**, 768–784 (2018).

36. V. Braun, A. C. Ratliff, H. Celia, S. K. Buchanan, Energization of outer membrane transport by the ExbB ExbD molecular motor. *J. Bacteriol.* **205**, e0003523 (2023).

37. T. Lithgow, C. J. Stubenrauch, M. P. H. Stumpf, Surveying membrane landscapes: A new look at the bacterial cell surface. *Nat. Rev. Microbiol.* **21**, 502–518 (2023).

38. A. Rocker, J. A. Lacey, M. J. Belousoff, J. J. Wilksch, R. A. Strugnell, M. R. Davies, T. Lithgow, Global trends in proteome remodeling of the outer membrane modulate antimicrobial permeability in *Klebsiella pneumoniae*. *MBio* **11**, e00603-20 (2020).

39. R. A. Dunstan, R. S. Bamert, K. S. Tan, U. Imbulgoda, C. K. Barlow, G. Taiaroa, D. J. Pickard, R. B. Schittenhelm, G. Dougan, F. L. Short, T. Lithgow, Epitopes in the capsular polysaccharide and the porin OmpK36 receptors are required for bacteriophage infection of *Klebsiella pneumoniae*. *Cell Rep.* **42**, 112551 (2023).

40. I. R. Vetter, M. W. Parker, A. D. Tucker, J. H. Lakey, F. Pattus, D. Tsernoglou, Crystal structure of a colicin N fragment suggests a model for toxicity. *Structure* **6**, 863–874 (1998).

41. W. Vollmer, B. Joris, P. Charlier, S. Foster, Bacterial peptidoglycan (murein) hydrolases. *FEMS Microbiol. Rev.* **32**, 259–286 (2008).

42. W. Vollmer, H. Pilsl, K. Hantke, J. V. Höltje, V. Braun, Pesticin displays muramidase activity. *J. Bacteriol.* **179**, 1580–1583 (1997).

43. S. I. Patzer, R. Albrecht, V. Braun, K. Zeth, Structural and mechanistic studies of pesticin, a bacterial homolog of phage lysozymes. *J. Biol. Chem.* **287**, 23381–23396 (2012).

44. L. Micenková, J. Bosák, J. Kucera, M. Hrala, T. Dolejšová, O. Šedo, D. Linke, R. Fišer, D. Šmajs, Colicin Z, a structurally and functionally novel colicin type that selectively kills enteroinvasive *Escherichia coli* and *Shigella* strains. *Sci. Rep.* **9**, 11127 (2019).

45. Y. Nishimura, T. Yoshida, M. Kuronishi, H. Uehara, H. Ogata, S. Goto, ViPTree: The viral proteomic tree server. *Bioinformatics* **33**, 2379–2380 (2017).

46. K. E. Holt, T. V. Thieu Nga, D. P. Thanh, H. Vinh, D. W. Kim, M. P. Vu Tra, J. I. Campbell, N. V. Hoang, N. T. Vinh, P. V. Minh, C. T. Thuy, T. T. Nga, C. Thompson, T. T. Dung, N. T. Nhu, P. V. Vinh, P. T. Tuyet, H. L. Phuc, N. T. Lien, B. D. Phu, N. T. Ai, N. M. Tien, N. Dong, C. M. Parry, T. T. Hien, J. J. Farrar, J. Parkhill, G. Dougan, N. R. Thomson, S. Baker, Tracking the establishment of local endemic populations of an emergent enteric pathogen. *Proc. Natl. Acad. Sci. U.S.A.* **110**, 17522–17527 (2013).

47. L. P. Nedialkova, R. Denzler, M. B. Koeppel, M. Diehl, D. Ring, T. Wille, R. G. Gerlach, B. Stecher, Inflammation fuels colicin Ib-dependent competition of *Salmonella* serovar Typhimurium and *E. coli* in *Enterobacterial* blooms. *PLOS Pathog.* **10**, e1003844 (2014).

48. E. Denkovskienė, Š. Paškevičius, A. Misiūnas, B. Stočkūnaitė, U. Starkevič, A. Vitkauskienė, S. Hahn-Löbmann, S. Schulz, A. Giritch, Y. Gleba, A. Ražanskienė, Broad and efficient control of *Klebsiella* pathogens by peptidoglycan-degrading and pore-forming bacteriocins klebicins. *Sci. Rep.* **9**, 15422 (2019).

49. X. Zhao, W. Wang, X. Zeng, R. Xu, B. Yuan, W. Yu, M. Wang, R. Jia, S. Chen, D. Zhu, M. Liu, Q. Yang, Y. Wu, S. Zhang, J. Huang, X. Ou, D. Sun, A. Cheng, Klebicin E, a pore-forming bacteriocin of *Klebsiella pneumoniae*, exploits the porin OmpC and the Ton system for translocation. *J. Biol. Chem.* **300**, 105694 (2024).

50. N. V. Ravin, A. N. Svarchevsky, G. Dehò, The anti-immunity system of phage-plasmid N15: Identification of the antirepressor gene and its control by a small processed RNA. *Mol. Microbiol.* **34**, 980–994 (1999).

51. E. Pfeifer, J. A. Moura de Sousa, M. Touchon, E. P. C. Rocha, Bacteria have numerous distinctive groups of phage-plasmids with conserved phage and variable plasmid gene repertoires. *Nucleic Acids Res.* **49**, 2655–2673 (2021).

52. R. Wolkowicz, M. Schaechter, What makes a virus a virus? *Nat. Rev. Microbiol.* **6**, 643 (2008).

53. P. Bourrat, P. E. Griffiths, Multispecies individuals. *Hist. Philos. Life Sci.* **40**, 33 (2018).

54. A. S. Lang, O. Zhaxybayeva, J. T. Beatty, Gene transfer agents: Phage-like elements of genetic exchange. *Nat. Rev. Microbiol.* **10**, 472–482 (2012).

55. I. Karaliute, R. Ramonaite, J. Bernatoniene, V. Petrikaite, A. Misiunas, E. Denkovskiene, A. Razanskiene, Y. Gleba, J. Kupcinskas, J. Skieceviciene, Reduction of gastrointestinal tract colonization by *Klebsiella quasipneumoniae* using antimicrobial protein KvarIa. *Gut Pathog.* **14**, 17 (2022).

56. Y. Perez-Riverol, J. Bai, C. Bandla, D. García-Seisdedos, S. Hewapathirana, S. Kamatchinathan, D. J. Kundu, A. Prakash, A. Frericks-Zipper, M. Eisenacher, M. Walzer, S. Wang, A. Brazma, J. A. Vizcaíno, The PRIDE database resources in 2022: A hub for mass spectrometry-based proteomics evidences. *Nucleic Acids Res.* **50**, D543–D552 (2022).

57. Y. Ueno, M. Arita, T. Kumagai, K. Asai, Processing sequence annotation data using the Lua programming language. *Genome Inform.* **14**, 154–163 (2003).

58. R. R. Wick, Filtlong, version 0.2.1, Github (2021); https://github.com/rrwick/Filtlong.

59. R. R. Wick, L. M. Judd, C. L. Gorrie, K. E. Holt, Unicycler: Resolving bacterial genome assemblies from short and long sequencing reads. *PLOS Comput. Biol.* **13**, e1005595 (2017).

60. T. Seemann, Prokka: Rapid prokaryotic genome annotation. *Bioinformatics* **30**, 2068–2069 (2014).

61. C. Camacho, G. Coulouris, V. Avagyan, N. Ma, J. Papadopoulos, K. Bealer, T. L. Madden, BLAST+: Architecture and applications. *BMC Bioinf.* **10**, 421 (2009).

62. A. W. Jenney, A. Clements, J. L. Farn, O. L. Wijburg, A. McGlinchey, D. W. Spelman, T. L. Pitt, M. E. Kaufmann, L. Liolios, M. B. Moloney, S. L. Wesselingh, R. A. Strugnell, Seroepidemiology of *Klebsiella pneumoniae* in an Australian tertiary hospital and its implications for vaccine development. *J. Clin. Microbiol.* **44**, 102–107 (2006).

63. C. L. M. Gilchrist, Y.-H. Chooi, clinker & clustermap.js: Automatic generation of gene cluster comparison figures. *Bioinformatics* **37**, 2473–2475 (2021).

64. L. Zimmermann, A. Stephens, S.-Z. Nam, D. Rau, J. Kübler, M. Lozajic, F. Gabler, J. Soding, A. N. Lupas, V. Alva, A completely reimplemented MPI Bioinformatics Toolkit with a new HHpred server at its core. *J. Mol. Biol.* **430**, 2237–2243 (2018).

65. M. Mirdita, K. Schütze, Y. Moriwaki, L. Heo, S. Ovchinnikov, M. Steinegger, ColabFold: Making protein folding accessible to all. *Nat. Methods* **19**, 679–682 (2022).

66. L. Holm, Dali server: Structural unification of protein families. *Nucleic Acids Res.* **50**, W210–W215 (2022).

67. E. F. Pettersen, T. D. Goddard, C. C. Huang, E. C. Meng, G. S. Couch, T. I. Croll, J. H. Morris, T. E. Ferrin, UCSF ChimeraX: Structure visualization for researchers, educators, and developers. *Protein Sci.* **30**, 70–82 (2021).

68. A. J. Page, C. A. Cummins, M. Hunt, V. K. Wong, S. Reuter, M. T. G. Holden, M. Fookes, D. Falush, J. A. Keane, J. Parkhill, Roary: Rapid large-scale prokaryote pan genome analysis. *Bioinformatics* **31**, 3691–3693 (2015).

69. A. J. Page, B. Taylor, A. J. Delaney, J. Soares, T. Seemann, J. A. Keane, S. R. Harris, SNP-sites: Rapid efficient extraction of SNPs from multi-FASTA alignments. *Microb. Genom.* **2**, e000056 (2016).

70. M. N. Price, P. S. Dehal, A. P. Arkin, FastTree 2 – Approximately maximum-likelihood trees for large alignments. *PLOS ONE* **5**, e9490 (2010).

71. I. Letunic, P. Bork, Interactive Tree Of Life (iTOL) v5: An online tool for phylogenetic tree display and annotation. *Nucleic Acids Res.* **49**, W293–W296 (2021).

72. Gatan Inc., DigitalMicrograph Software (DM3) (2024); www.gatan.com/products/tem-analysis/gatan-microscopy-suite-software.

73. M. HaileMariam, R. V. Eguez, H. Singh, S. Bekele, G. Ameni, R. Pieper, Y. Yu, S-Trap, an ultrafast sample-preparation approach for shotgun proteomics. *J. Proteome Res.* **17**, 2917–2924 (2018).

74. S. J. Humphrey, O. Karayel, D. E. James, M. Mann, High-throughput and high-sensitivity phosphoproteomics with the EasyPhos platform. *Nat. Protoc.* **13**, 1897–1916 (2018).

75. J. Cox, M. Y. Hein, C. A. Luber, I. Paron, N. Nagaraj, M. Mann, Accurate proteome-wide label-free quantification by delayed normalization and maximal peptide ratio extraction, termed MaxLFQ. *Mol. Cell. Proteomics* **13**, 2513–2526 (2014).

76. J. Cox, M. Mann, MaxQuant enables high peptide identification rates, individualized p.p.b.-range mass accuracies and proteome-wide protein quantification. *Nat. Biotechnol.* **26**, 1367–1372 (2008).

77. A. D. Shah, R. J. A. Goode, C. Huang, D. R. Powell, R. B. Schittenhelm, LFQ-Analyst: An easy-to-use interactive web platform to analyze and visualize label-free proteomics data preprocessed with MaxQuant. *J. Proteome Res.* **19**, 204–211 (2020).

78. R. A. Dunstan, I. D. Hay, T. Lithgow, Defining membrane protein localization by isopycnic density gradients. *Methods Mol. Biol.* **1615**, 81–86 (2017).

79. C. T. Webb, J. Selkrig, A. J. Perry, N. Noinaj, S. K. Buchanan, T. Lithgow, Dynamic association of BAM complex modules includes surface exposure of the lipoprotein BamC. *J. Mol. Biol.* **422**, 545–555 (2012).

80. C. J. Stubenrauch, R. S. Bamert, J. Wang, T. Lithgow, A noncanonical chaperone interacts with drug efflux pumps during their assembly into bacterial outer membranes. *PLOS Biol.* **20**, e3001523 (2022).

81. A. M. Bolger, M. Lohse, B. Usadel, Trimmomatic: A flexible trimmer for Illumina sequence data. *Bioinformatics* **30**, 2114–2120 (2014).

82. T. Seemann, Snippy: Fast bacterial variant calling from NGS reads, version 4.6.0, Github (2015); https://github.com/tseemann/snippy.

83. G. J. McKenzie, N. L. Craig, Fast, easy and efficient: Site-specific insertion of transgenes into enterobacterial chromosomes using Tn*7* without need for selection of the insertion event. *BMC Microbiol.* **6**, 39 (2006).

84. I. R. Lee, J. S. Molton, K. L. Wyres, C. Gorrie, J. Wong, C. H. Hoh, J. Teo, S. Kalimuddin, D. C. Lye, S. Archuleta, K. E. Holt, Y.-H. Gan, Differential host susceptibility and bacterial virulence factors driving *Klebsiella* liver abscess in an ethnically diverse population. *Sci. Rep.* **6**, 29316 (2016).

85. M. M. C. Lam, K. L. Wyres, S. Duchêne, R. R. Wick, L. M. Judd, Y.-H. Gan, C.-H. Hoh, S. Archuleta, J. S. Molton, S. Kalimuddin, T. H. Koh, V. Passet, S. Brisse, K. E. Holt, Population genomics of hypervirulent *Klebsiella pneumoniae* clonal-group 23 reveals early emergence and rapid global dissemination. *Nat. Commun.* **9**, 2703 (2018).

86. K. M. Wu, L. H. Li, J. J. Yan, N. Tsao, T. L. Liao, H. C. Tsai, C. P. Fung, H. J. Chen, Y. M. Liu, J. T. Wang, C. T. Fang, S. C. Chang, H. Y. Shu, T. T. Liu, Y. T. Chen, Y. R. Shiau, T. L. Lauderdale, I. J. Su, R. Kirby, S. F. Tsai, Genome sequencing and comparative analysis of *Klebsiella pneumoniae* NTUH-K2044, a strain causing liver abscess and meningitis. *J. Bacteriol.* **191**, 4492–4501 (2009).
